# Childhood Demographics and Socioeconomic Conditions Predict Reproduction 15 Years Later

**DOI:** 10.1177/14747049261432881

**Published:** 2026-04-09

**Authors:** Vinícius Betzel Koehler, M.D. Rutherford

**Affiliations:** 1 Department of Psychology, Neuroscience & Behaviour, 3710 McMaster University, Hamilton, Canada

**Keywords:** life history theory, psychosocial acceleration theory, harshness, unpredictability, reproduction, census, indigenous people

## Abstract

Psychosocial acceleration theory (PAT) posits that experiencing harsh and unpredictable environments during childhood cues the development of earlier and more frequent reproductive events. However, this developmental association has not been tested in large human populations. Visible minorities and Indigenous people are also subjected to harsher and more unpredictable circumstances than the general population. These circumstances might not be detected in common measures used in PAT literature. In four studies, we tested whether an exploratory analytical approach using measures of harshness and unpredictability from census data of a high-income country (Canada) would be relevant predictors of measures of reproduction 15 years later, whether the proportion of Indigenous people and of visible minorities are relevant predictors, and tested methodological and statistical assumptions and limitations of this approach. Following PAT assumptions, we hypothesize that higher rates of harshness and unpredictability will be associated with higher rates of reproduction. Results were mixed in offering support or working against PAT claims. A higher percentage of children in low-income households was predictive of a higher percentage of single-parent households in Canadian census divisions. However, measures indicative of lower access to resources and unpredictable parental availability were negatively predictive of reproduction outcomes. A higher percentage of Indigenous people was also predictive of a larger family size of single parents. Findings can help inform public policies around early pregnancy and family planning.

## Introduction

### Life History Theory

Life history theory proposes that the optimal allocation of resources to different functions varies among species and across diverse environmental contexts (Del Giudice et al., 2015; Stearns, 1992). Resources such as energy and time are limited, and an organism has to allocate these resources across many functions. An organism needs energy to maintain its body functioning or to grow its body, depending on its time of development. It also needs to seek food and mates; build or acquire resources (e.g., find or defend territory, a nest or mound, tools); heal from wounds or fend off pathogens; reproduce; and, for many species, invest some resources in offspring.

Depending on the environment and niche, different patterns of investments are more adaptive (Ellis et al., 2009). For example, for a species under considerable predation, investing in acquiring resources is unlikely to pay off. It may be more adaptive to invest in reaching sexual maturity earlier and reproducing faster, reducing the chances of being preyed upon before passing on its genes. Differences in the optimal level of investment for each body function at each time of development lead to the development of different life history strategies (LHS). Life history theory had its origins in biology (LHT-B), and it started by describing differences between species (Del Giudice, 2009; Stearns, 1992), but it has also been used to describe differences within species (Albaladejo-Robles et al., 2023; Stearns et al., 2008; Stone et al., 2023), including humans (Del Giudice & Belsky, 2010; Dinh et al., 2022), which started the related field of life history theory in evolutionary psychology (LHT-P; Nettle & Frankenhuis, 2020).

A considerable amount of research in LHT-P has been focused on assessing how environments high in harshness—understood as extrinsic mortality—and unpredictability—understood as random variation of harshness—can influence humans’ LHS (Ellis et al., 2009; Simpson et al., 2012; Webster et al., 2014; Xu et al., 2018). The different LHS have been assumed to lie on a continuum from slow to fast. People on the faster end of this LHS continuum would favor investments in earlier and frequent reproduction, whereas people on the slower end would delay investments in reproduction to favor investments in growth. A faster LHS would be more adaptive in harsh and unpredictable environments because an individual would be less certain of their future chances of reproduction.

Density levels are also hypothesized to have an effect on LHS (Stearns, 1992). Low-density environments would favor a faster LHS because those adopting this strategy would have higher chances of occupying the area and benefit from the available resources. On the other hand, higher density environments would favor a slower LHS because offspring would have to compete with others to acquire resources (Del Giudice et al., 2015; Ellis et al., 2009; Sear, 2020). The effect of density on LHS was also hypothesized to be dependent on the levels of harshness and unpredictability. For example, low-density would particularly increase the fitness of those adopting a faster LHS in relatively stable and resourceful environments. LHT-P research, however, has mostly ignored the effect of density on LHS (Stearns & Rodrigues, 2020; Volk, 2025).

Within LHT-P, Belsky et al. (1991) have proposed the psychosocial acceleration theory (PAT). PAT posits that cues of harshness and unpredictability early in childhood can trigger a series of developmental milestones, including earlier puberty (Webster et al., 2014; Xu et al., 2018) and earlier and more frequent reproductive events (Dinh et al., 2022; Wilson & Daly, 1997). Parental absence—usually the father—has been argued to be the best predictor of this developmental shift (Ellis et al., 2003; Hartman et al., 2018; Webster et al., 2014), and the first 7 years of life would be the critical time of development to shape one's LHS (Ellis et al., 2003; Simpson et al., 2012; Webster et al., 2014; Xu et al., 2018).

LHT-P has been under criticism. One such criticism is that it has considerably departed from LHT-B, which uses more formal modeling and more specific predictions (Nettle & Frankenhuis, 2020). The fast–slow continuum of LHS has been abandoned in LHT-B. Research in the field of biology has focused on how specific environments would favor different life history traits and not a suite of traits as it has been studied in psychology (Nettle & Frankenhuis, 2020).

The existence of LHS—understood as a suite of correlated behaviors—and the utility of LHS to explain behaviors (Sear, 2020; Stearns & Rodrigues, 2020) or the validity of PAT assumptions has been questioned (Volk, 2023, 2025). In a meta-analysis, Webster et al. (2014) noted studies with bigger sample sizes found a small association between father absence and menarche. Some studies have also failed to find results that support LHT-P and PAT assumptions (e.g., Nolin & Ziker, 2016; Richardson et al., 2020, 2024; Wells et al., 2019).

The definitions or measures of harshness and unpredictability have also been criticized. For example, unpredictability lacks a clear statistical definition (Young et al., 2020), such as whether it means a sudden change point, high variance of harshness, or high autocorrelation with harshness. Would only unpredictability that increases harshness lead to the development of faster LHS, or would unpredictability that decreases the mean level of harshness also lead to a faster LHS? The definition of harshness is the level of extrinsic mortality in the population, but the connection of this definition and the usual measures of harshness used in LHT-P and PAT literature (e.g., socioeconomic status) are still not clear, especially in modern environments or high-income countries (Stearns & Rodrigues, 2020; Volk, 2023, 2025). Many have argued that LHT-P and PAT should go into considerable revision, and in doing so, researchers should aim to harmonize the theories in psychology with those in evolutionary biology (Nettle & Frankenhuis, 2020; Sear, 2020; Stearns & Rodrigues, 2020; Volk, 2025).

In several studies, PAT research has used socioeconomic indicators as a measure of resource access and therefore a measure of harshness (Copping & Campbell, 2015; Hartman et al., 2018; Simpson et al., 2012) and measures of parental transitions (e.g., household configuration change and employment change) and geographical moves as measures of unpredictability (Belsky et al., 2012; Young et al., 2020). Earlier or faster reproduction has been measured as time of menarche (because menarch is a direct and usually memorable puberty marker,Xu et al., 2018) and as first time having sex or first time having children (Ellis et al., 2003; Webster et al., 2014).

Considering the developmental aspect of PAT (i.e., exposure to certain environments in the first 7 years of life shaping a series of reproductive milestones), a longitudinal design would be ideal to test such a hypothesis. The measures that are often used in PAT (i.e., socioeconomic status, employment, geographic moves, household configuration, and fertility) are similar to measures usually present in censuses and other governmental reports (Statistics Canada, 2024). Censuses and other governmental reports are also often publicly available, which makes them an invaluable asset for research (Johnston, 2017; Trzesniewski et al., 2011). Leveraging these data is particularly true for research exploring PAT claims (Copping, 2017) because the periodicity of this governmental data fits well with PAT developmental description. Studies that use large sample sizes or large populations, and more importantly, studies that offer cross-cultural findings are also especially valuable for evolutionary psychology because they are supportive that such findings are reflective of an adaptation or of an evolved mechanism instead of some other phenomenon (Buss, 2024).

The use of census data, however, has not been well explored in PAT research. Most PAT research has either used convenience samples (Sear, 2020) or longitudinal surveys from a few samples (Kelly et al., 2017; Magnus et al., 2018; Young et al., 2020). To the best of our knowledge, research testing LHT-P hypotheses and using census or entire population data has only been conducted in England and Wales (Copping et al., 2013; Copping & Campbell, 2015).

### Indigenous People and Visible Minorities in Canada

First, we would like to acknowledge that the use of *Indigenous people* is not sufficient to reference the heterogeneity of the cultural groups expressed by this term (Statistics Canada, 2017). In Canada, this term refers to First Nations, Métis, and Inuit communities, each with their own cultural identity, governance, and history. The choice to use *Indigenous people* in this study aims to reflect the terminology adopted by Statistics Canada (*Dictionary, Census of Population, 2021*, 202*3*), which reported the counts of *Aboriginal ancestry population* in 2006 and *Indigenous identity* in 2021 (Statistics Canada, 2024).

Indigenous people are more likely to be subject to harsh circumstances compared to non-Indigenous people in Canada (*Honouring the Truth, Reconciling for the Future*, 201*5*). Colonial history, confinement of its culture and ways of living to “reservations,” and structural inequities (Neu & Graham, 2006; Romaniuk, 2008) all cause Indigenous people to experience harsher environments, on average. According to LHT-P rationale, these circumstances may be part of the cause for Indigenous people to be younger (Statistics Canada, 2023), faster growing (Statistics Canada, 2017), and to have disproportionately high rates of teenage pregnancy (Reading & Wien, 2009) than non-Indigenous people.

Visible minorities are also socially disadvantaged in Canada. *Visible minority* is defined by Statistics Canada as “persons, other than Aboriginal peoples, who are non-Caucasian in race or non-white in color” (*Dictionary, Census of Population, 2021*, 202*3*, p. 169). They are often targets of discrimination and trauma (Williams et al., 2022), including unequal access to employment (Henry et al., 1985; Intungane et al., 2024), and healthcare (Husbands et al., 2022). Similarly to the case of Indigenous people, immigrants—who are often from non-White ethnicities—also have more children than the nonimmigrant population (Bélanger et al., 2006). These particularly harsh and unpredictable circumstances faced by Indigenous people and visible minorities may not be captured in common PAT research.

### Current Study

This study uses an exploratory analytical approach to assess five research questions. Our first research question asks whether indicators of harshness and unpredictability, as informed by PAT, present in the census successfully predict reproduction indicators 15 years later. This research question is intended to test if the general claim of PAT (i.e., early harshness and unpredictability shapes future reproduction) is also present in geographical data (i.e., describing entire populations).

The second research question is intended to inform us about the likelihood that population mobility across time is affecting the results. We are interested in knowing whether a model built using either a smaller or larger geography level will better predict reproduction 15 years later. Dissemination areas are the smallest geography level reported by the Canadian census, and it is an area that comprehends an average of 400 to 700 people, whereas census divisions are deemed the most stable geography level (*Dictionary, Census of Population, 2021*, 202*3*) that is usually composed of neighboring municipalities. Please see the description of these geography levels in the Method section.

The third and fourth research questions test the developmental aspect of PAT. The third research question asks which model will better predict reproduction: a model using data in the correct timeline (i.e., harshness and unpredictability predicting reproduction 15 years later) or a model using data in an inverted timeline (i.e., harshness and unpredictability predicting reproduction 15 years prior). This research question is intended to test if results are likely to be describing a developmental and longitudinal phenomenon or just some statistical association across time. The fourth research question tests whether the model will perform better in geographies with higher proportions of children. This is also intended to test the developmental assumption of PAT but taking into consideration the critical period (first 7 years of life) of exposure to harshness and unpredictability to shape future reproduction.

Our final research question asks whether the proportions of Indigenous people and of visible minorities will be relevant predictors of reproduction. Indigenous people and visible minorities are exposed to particularly harsh and unpredictable circumstances that may not be usually captured in the measures commonly used in PAT research. We ask then if the proportions of these two populations will be relevant predictors of reproduction even when indicators of harshness and unpredictability are included in the model.

Following PAT rationale, we hypothesize that the model will be successful at explaining a good amount of variance of reproduction indicators in Canada. However, we are not sure whether the model built with smaller geography or larger geography data will perform better. Data from smaller geographies will yield higher statistical power and more variance because of their smaller convergence to mean values. Larger geographies will be more stable and less susceptible to noise due to migration.

We hypothesize that harsher and more unpredictable measures will be predictive of higher reproduction rates 15 years later, but that such an association will not hold in a model with an inverted timeline. We finally hypothesize that the model using data from geographies with a higher proportion of children and with a higher proportion of Indigenous people and visible minorities will predict reproduction better than the model using data from geographies with a smaller proportion of these groups.

The studies in this manuscript will use geographic-level data (e.g., the rates or averages of people in a municipality that fall under a certain criterion) to assess the common claims and assumptions of PAT, a theory that aims to explain individual-level phenomena. The findings in this manuscript must be, therefore, interpreted with caution. For example, the discussion of the findings here relies on the assumption that most of the population of a given geography remained in that same geography across time. This assumption is not necessarily met. We discuss and assess the likelihood that this assumption is violated in the article.

Another issue is that associations that we find at the geographic level may not be present at the individual level. Confounding variables or associations that exist at the geographic level but that are actually describing two different populations limit the inferences that can be drawn from our findings. Thus, our focus will be on the prediction and detection of patterns, rather than on causal or individual-level inferences.

## Methods

### Data Selection and Transformation

We accessed census data through the Canadian Census Analyser (Statistics Canada, 2024) available through the university library. Data were extracted from 52,973 dissemination areas (DA) and 288 census divisions (CD) in the 2006 Census and 57,936 DA and 293 CD in the 2021 Census. A dissemination area is a “small, relatively stable geographic unit composed of one or more adjacent dissemination blocks with an average population of 400 to 700 persons” (*Dictionary, Census of Population, 2021*, 202*3*, p. 86), and they cover all Canadian territory, whereas CD are larger geographies composed of groups “of neighbouring municipalities joined together […]” and “are the most stable administrative geographic areas” (*Dictionary, Census of Population, 2021*, 202*3*, p. 68) next to provinces or territories. After merging the 2006 and the 2021 data frames, 48,867 DA and 286 CD were left. This loss in the number of cases is due to geographical redefinition or recoding in this period.

We extracted 120 variables that we considered of any relevance to the research question from the 2006 Census and 235 variables from the 2021 Census. These variables comprised information about age and sex, family and dwelling characteristics, income, immigration, labor, education, and Indigenous and visible minorities. These variables were assessed and thematically grouped by the first author to create the factors used in this analysis. The variables were selected based on their relevance to the research questions. The first iteration of the model used 40 variables. Table 1 describes the variables and the factors used in the model, and *Variables* in supplementary materials provides a full list of variables extracted and how they were classified. All of the materials, raw data, and transformed data for all the studies are openly available on OSF: https://osf.io/45vru/overview?view_only=47a22c13dcc14a8da5b09dd676ddca87. This study was not preregistered.

**Table 1.** Variables Fed Into the First Models in Study 1.

LHT Concept	Usual Measure in LHT	Factor Loaded in 1st Iteration	Variables	Final Model
Harshness	SES measures	Income	Median family income	DA
			Prevalence children 6 years of age or less living with low income before tax	CD
		Lack of resources	Occupied private dwellings needing minor repairs	DA
			Occupied private dwellings needing major repairs	DA
			Tenant occupied households spending more than 30% on rent	CD; DA
			Employed labor force 15 years of age and over using public transit	
		Low schooling	Population 25–64 with no certificate, diploma or degree	
Unpredictability	Parental transitions	Female lone parent	Female lone parent	
			Median of female lone-parent income	
			Percentage of female lone-parent income coming from other sources (i.e., neither employment nor government transfers)	
		Male lone parent	Male lone parent	
			Median of male lone-parent income	
			Percentage of male lone-parent income coming from other sources (i.e., neither employment nor government transfers)	
		Separated	Divorced	DA
			Widowed	DA
			Separated, but still legally married	
	Parental occupation transitions	Precariously labor	Unemployment rate of population 25 years and over	CD
			People 15 years and over who worked in different census subdivision	
			Unemployment rate of population 15 years and over with children at home	
			People 15 years and over self-employed (unincorporated) without paid help	
			People 15 years and over who worked part year or part time	
	Geographical transitions	Migrants and speaking foreign languages	Movers 1 year ago	
			Movers 5 years ago	
			Neither English nor French as first official language spoken	
			Nonofficial language spoken in single responses	
			English and nonofficial language in multiple responses	
			French and nonofficial language in multiple responses	
-	-	Indigenous	Total aboriginal ancestry population	CD
		Visible minority	Total visible minority population	DA
			Nonofficial language spoken in single responses	DA
-	-	Young children	Age group 0–4 years of age	CD; DA
			Age group 5–9 years of age	DA
Reproduction	Age of menarche, number of partners, number of children, and interbirth interval	Frequent reproduction	Average size of families	CD; DA
			Average number of children in families with children	CD; DA
			Families with 4 persons	CD; DA
			Families with 5 or more persons	CD; DA
		Single parenting	Average family size of one-parent families	CD
		Big families	Private households with 4 persons	
			Private households with 5 or more persons	
		Recent reproduction	Age group 0–4 years of age	CD

*Note*. Harshness and unpredictability variables collected from census 2006 and reproduction variables collected from 2021. Final model indicates whether the variable was a relevant and significant in the models using CD or DA sample.

CD=census division; DA= dissemination area; LHT= Life history theory.

We assessed NAs next with a 5% cutoff established (i.e., if more than 5% of the values were NAs, the variable would be excluded), but no variables met such cutoff. Most of the variables were right skewed (i.e., with distribution close to 0). Aiming for a distribution closer to a normal distribution, all variables were square-root and log transformed and visually assessed with boxplots. The first author visually inspected the boxplots for each variable (original, square root-, and log-transformed) and selected the data transformation that resulted in a distribution closer to a normal distribution. This decision was only made when variables had a notable difference in distribution (i.e., the median was closer to the center of the quartiles, whiskers of relatively equal lengths, and fewer outliers). Whenever differences were not notable, the order of preference was (1) variable with no transformation; (2) variable with square root transformation; and (3) variable with log transformation (Supplemental Table S1).

In the transformation process, we identified that four variables had more than 75% of zeros (median male lone parent income, percentage of male lone parent income coming from other sources, prevalence of low income, and people speaking French and a nonofficial language). These variables were removed from the model because they could distort the relationship between variables. Finally, we defined outliers in this data frame as cases with a z-score above the absolute value of 3 on any variable. Outlier cases were also removed. The data frame used for the model had 38 variables and 39,481 cases in the DA sample and 240 cases in the CD sample (see Table 1).

### Partial Least Squares Structural Equation Modeling

Partial least squares structural equation modeling (PLS-SEM) is an exploratory and predictive analytical approach focused on explaining the variance in the dependent variables (Hair et al., 2021). It does so by combining a measurement model (factor analysis) and a structural model (path analysis), and it relies on several statistics for the evaluation of the model's quality (Hair et al., 2022). PLS-SEM is a nonparametric analysis, and it is robust with formative factors (Hair et al., 2021). Formative factors are a group of items that are understood to be forming the factor. PLS-SEM also allows for single-item “factors,” which is a good advantage when working with secondary data. These make PLS-SEM well suited for this study.

Large samples generate lower *p*-values. Considering the dissemination area sample size in this study, it is likely that we would interpret results in the analysis as significant merely due to the sample size. Because the census is the best description of a population, any obtained result is descriptive of the Canadian population, regardless of statistical significance. In addition, PLS-SEM has been criticized for how it calculates statistical significance (Rönkkö et al., 2015), but many evaluations of a model's quality in PLS-SEM are assessed using *p*-values. To deal with this issue, we established a significance of *α* ≤ .01 (*t* ≥ 2.576) for our analyses, and we focused more on confidence intervals not being zero and on thresholds recommended by Hair et al. (2019, 2021) for accepting the measurement and structural models. A criterion in which we differed slightly from Hair et al. (2019, 2021) was the use of the following R^2^ parameters: < .2 = negligible; from .2 to .5 = weak; from .5 to .7 = moderate; > .7 = strong (Nau, 2020).

### Model

All the models in this article had predictors set as formative factors and outcomes set as reflective factors. The biggest change between these two is whether the items that are making that factor are understood to form or to reflect their factor. The items are understood to describe the factors in a formative factor (e.g., one of our predictors, *Lack of resources,* being formed or being described as people living in households in need of minor repairs, major repairs, or spending more than 30% of their income on rent). On the other hand, outcomes were set as reflective factors, in which the items are understood to reflect or to be caused by the factor (e.g., a faster LHS causing people to have larger families with two or more children; Hair et al., 2021).

**Figure 1.**
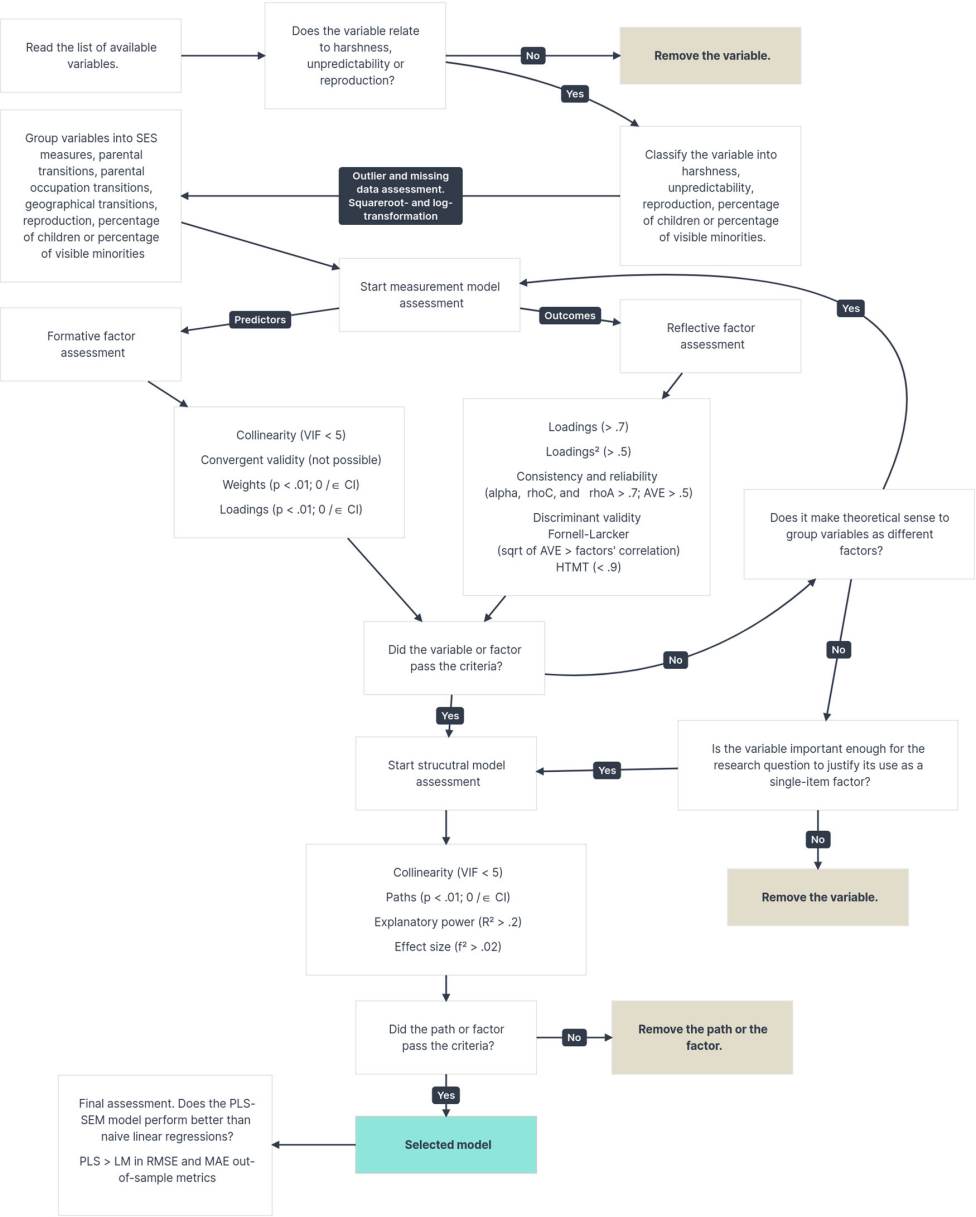
Decision-making process in the PLS-SEM analysis.

*Note.* AVE=average variance extracted; HTMT=heterotrait-monotrait ratio; PLS-SEM=partial least squares structural equation modeling.

Following Hair et al.’s (2019, 2021) guidelines, we assessed the reflective factors loadings (>.7), indicators reliability (loading^2^ > .05), internal consistency (α, ρC, ρA > .7) and reliability (AVE > .5), and discriminant validity (HTMT < .9 and Fornell-Larcker criterion, in which the constructs correlations should be lower than the square root of the AVE). Formative factors were assessed with collinearity (VIF < 5), and weights and loadings for significance and relevance of indicators. Convergent validity analysis was not possible because there would not be an alternative measure, nor would it be possible to resample participants who responded to the census. Finally, we assessed collinearity (VIF < 5), relevance and significance of paths (bootstrapped *β* *≥* *|.1|*, *t* ≥ 2.576, and CI not including zero), and explanatory power (adjusted *R*^2^ > .2) in the structural model. Paths that were above criteria in these assessments and outcome variables with sufficient explanatory power were selected. We also assessed predictive power using a k-fold cross-validation model (k = 10) with root mean squared error (RMSE) out-of-sample between the PLS-SEM models and models using naïve linear regressions to check if the grouping into latent variables would outperform a path between observed variables. Figure 1 illustrates the steps and decision-making process in the analysis. See Supplemental materials “Building the model” for the full analytical report.

## Study 1: Are Dissemination Areas or Census Divisions the Best Geographical Level for Analysis?

In the first study, we aimed to build one model using DA and to build a second one using CD data. Our goal was to offer insight into the hypothesis of population mobility. We hypothesized that measures akin to the common measures of harshness and unpredictability used in research would predict measures of reproduction present in both datasets. We established an adjusted *R*^2^ > |0.1| difference to consider that the models are performing differently, but we did not have a specific hypothesis about whether the model using DA data would have a higher *R*^2^ than the one using CD data.

### Method

The method for this study followed the steps described in the methods section above. The data were transformed for both DA and CD because they were organized in two different data frames. See Table 1 for a list and description of the variables and factors used in the first iteration of the model.

### Results

**Figure 2.**
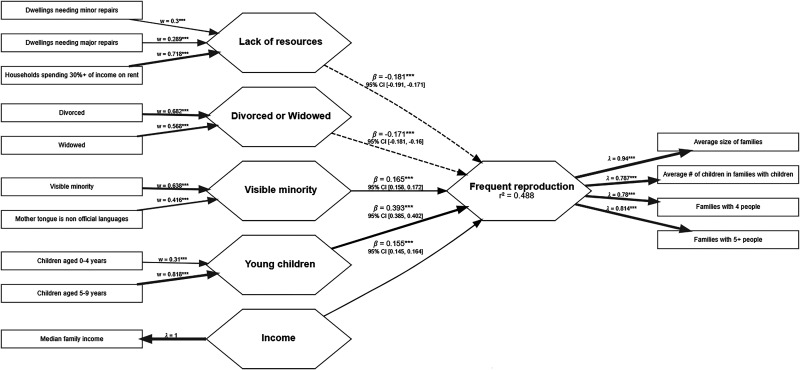
Proportion of young children, rates of visible minorities in the population, and socioeconomic factors predict reproduction in dissemination areas.

*Note*. Figure created in R using seminr package. Hexagons represent latent variables and rectangles represent items from census. Predictors are formative latent variables using census data in 2006 and outcomes are reflective latent variables using census data in 2021. DA = dissemination areas; CD = census divisions; W: variable's weights; λ: variable's loadings; β: path's beta coefficients; **: *p* ≤ .01, ***: *p* ≤ .001.

**Figure 3.**
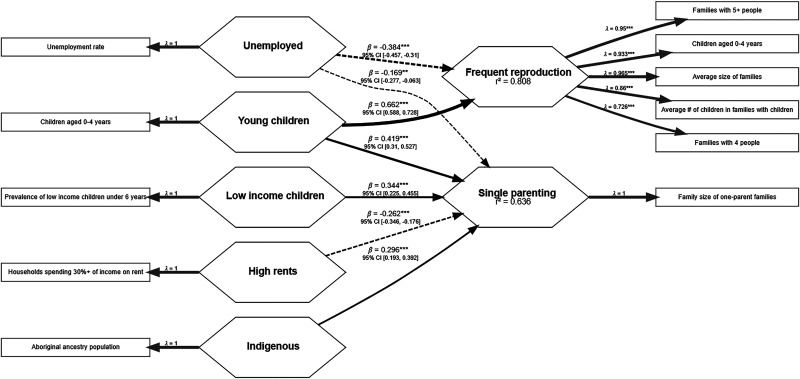
Proportion of young children, rates of indigeneity in the population, and socioeconomic factors predict reproduction in census divisions.

*Note*. *Note*. Figure created in R using seminr package. Hexagons represent latent variables and rectangles represent items from census. Predictors are formative latent variables using census data in 2006 and outcomes are reflective latent variables using census data in 2021. DA = dissemination areas; CD = census divisions; W: variable's weights; λ: variable's loadings; β: path's beta coefficients; **: *p* ≤ .01, ***: *p* ≤ .001.

Figure 2 illustrates the models built with DA and Figure 3 illustrates the model built with CD data. Rectangles represent the variables extracted from the census, and hexagons represent the factors the variables were loaded into. The arrows represent either the paths between factors or between variables and factors. Arrows pointing from variables to the factors represent formative factors, and arrows pointing from the factors to variables represent reflective factors. In the case of single-item factors, the arrows convey no meaning other than indicating that the factor is composed of that single variable. An arrow's width represents the path's strength, and dashed lines indicate a negative association. All the variables present in the models on both DA and CD samples were above the criteria of model quality. In the reflective model assessment, loadings were *Average size of families* = .97, *Average number of children in families with children* = .86, *Families with 4 people* = .73, *Families with 5 or more people* = .95, and *Children aged 0–4 years* = .93 on the CD model and *Average size of families* = .94, *Average number of children in families with children* = .79, *Families with 4 people* = .78, and *Families with 5 or more people* = .81 on the DA model. Indicator's reliability scores were *Average size of families* = .93, *Average number of children in families with children* = .74, *Families with 4 people* = .53, *Families with 5 or more people* = .90, and *Children aged 0–4 years* = .87 on the model using CD data; and *Average size of families* = .88, *Average number of children in families with children* = .62, *Families with 4 people* = .61, and *Families with 5 or more people* = .66 on the model using DA data. Internal consistency was high (α = .93, ρC = .95, ρA = .94 and α = .86, ρC = .90, ρA = .88), and reliability was supported by AVE values of .79 and .70 for the CD and DA models, respectively. Considering the confidence upper limit, the HTMT discriminant validity criterion did not pass the criterion between the variables of *Young children* and *Frequent reproduction* (.91) on the CD sample but passed the criteria on the DA sample. However, the correlations between variables were below the AVE on the assessment of the Fornell-Larcker criterion on both samples, which indicates they achieved discriminant validity. Table 2 reports the formative measurement model assessments of the DA model. Since all predictors in the CD models were single-item variables, the measurement model assessment of such variables is not applicable. Pearson's r correlation tables, means, and standard deviations of the variables used in the first iterations of both DA and CD models are included in the Supplemental materials.

**Table 2.** Formative Latent Variables Assessment of Dissemination Areas Model in Study 1.

Latent Variables		VIF	Weights	Loadings
Lack of resources				
	Dwellings needing minor repairs	1.14	0.30	0.58
	Dwellings needing major repairs	1.19	0.29	0.62
	Households spending 30%+ of income on rent	1.17	0.72	0.90
Divorced or Widowed				
	Divorced	1.08	0.68	0.84
	Widowed	1.08	0.57	0.75
Visible minority				
	Visible minority	2.68	0.64	0.97
	Mother tongue is non official languages	2.68	0.42	0.92
Young children				
	Children aged 0–4 years	1.27	0.31	0.69
	Children aged 5–9 years	1.27	0.82	0.96

*Note*. VIF: collinearity assessment. Bootstrapped weights and loadings were all significant (*p* < .01) and confidence intervals did not cross zero. Income variable was a single-item variable; therefore, no assessment was made.

Both models were not collinear and had statistically significant (*p* *<* *.01*) and relevant (*β* *≥* *|.1|)* predictors of *Frequent reproduction*. The explanatory power (*R*^2^) of *Frequent reproduction* in both samples was above the determined criteria. The explanatory power was weak (adjusted *R^2^* = .49) in the DA sample and strong (adjusted *R^2^* = .81) in the CD sample. The model built using the CD sample also moderately explained the variance of female single parenting (adjusted *R^2^* = .64), and it was a more parsimonious model, utilizing only five single-item variables. All of these support the hypothesis that the CD is a more stable geographic organization and is less affected by population mobility than the DA geographic organization. The CD data is, therefore, more suitable for the analyses in this manuscript. Mostly medium and large effect sizes were obtained on the CD model and small on the DA sample. Table 3 reports the structural model assessment of both models.

**Table 3.** Structural Model Assessment in Study 1.

Dissemination Areas Model			
Predictors	Frequent Reproduction		
	VIF	Paths	f^2^
Income	1.68	0.16	0.04
Lack of resources	1.87	− 0.18	0.03
Divorced or Widowed	1.91	− 0.17	0.03
Visible minority	1.07	0.17	0.06
Young children	1.27	0.39	0.21
Adjusted R^2^	.49		
**Census divisions model**			
**Predictors**	**Frequent reproduction**		
Unemployed	1.24	− 0.38	0.62
Young children	1.24	0.66	1.82
Adjusted R^2^	.81		
**Predictors**	**Single parenting**		
Low-income children	1.60	0.34	0.20
High rents	1.49	− 0.26	0.13
Unemployed	1.44	− 0.17	0.06
Indigenous	1.62	0.30	0.15
Young children	1.73	0.42	0.28
Adjusted R^2^	.64		

*Note*. VIF: collinearity assessment. Bootstrapped paths were all significant (*p* < .01) and confidence intervals did not cross zero.

A few distinctions are worth noting. On the CD sample, only the variables of young children aged 0 to 4 years (*Young children)* and the unemployment rate of people aged 25 and over (*Unemployed*) are relevant in predicting *Frequent reproduction*. However, *Young children* was negatively associated with *Frequent reproduction*, and *Unemployed* was negatively associated with both *Frequent reproduction* and *Single parenting*. Households spending more than 30% of their income on rent (*High rents*) was also a relevant and significant predictor but negatively associated with *Single parenting*. *Young children* is the same variable as one of the variables loading into *Frequent reproduction*, only separated by 15 years. On the DA samples, *Lack of resources* and *Divorced or Widowed* were negatively associated with *Frequent reproduction,* and median family income (*Income)* was positively associated with *Frequent reproduction*. All of these associations in both models were contrary to usual hypotheses of PAT. Therefore, the variables positively associated with *Frequent reproduction* were *Young children* and *Visible minority*. Differently from the model using CD samples, the variables composing the *Young children* factor were not included in the variables in the *Frequent reproduction* factor on the DA samples. *Visible minority* was a relevant predictor of *Frequent reproduction* on the DA samples, and *Indigenous* was a relevant predictor of the average family size of one-parent families (*Single parenting*).

On the CD sample, the effect sizes of the variables predicting *Frequent reproduction* were large, and the effect sizes of variables predicting *Single parenting* were small to medium. On the other hand, on the DA sample, the effect sizes of the variables predicting *Frequent reproduction* were mostly small, with the exception of the medium effect size of *Young children*. The RMSE out-of-sample metrics predictive values were lower on the naive linear model (CD: *Average size of families* = .06, *Average number of children in families with children* = .07, *Families with 4 people* = .51, *Families with 5 or more people* = .27, *Children aged 0–4 years* = .49, *Family size of one-parent families* = .07; DA: *Average size of families* = .05, *Average number of children in families with children* = .06, *Families with 4 people* = .30, *Families with 5 or more people* = .43) than on the PLS-SEM (CD: *Average size of families* = .06, *Average number of children in families with children* = .08, *Families with 4 people* = .55, *Families with 5 or more people* = .34, *Children aged 0–4 years* = .52, *Family size of one-parent families* = .08; DA: *Average size of families* = .05, *Average number of children in families with children* = .06, *Families with 4 people* = .32, *Families with 5 or more people* = .44). The MAE out-of-sample metrics predictive values were also lower than the PLS-SEM values. This indicates that the linear model performed better (i.e., its predictions resulted in lower errors) if such models were tested with unseen data.

### Discussion

The model on CD was the most parsimonious model (i.e., using the smallest number of predictive variables) and was able to predict more variables with both higher explanatory power and effect sizes. This suggests that using CD is the most reliable geographic level to predict *Frequent reproduction* in Canada. Indeed, CD is the most stable administrative geographic area (*Dictionary, Census of Population, 2021*, 202*3*) with yearly interprovincial migration rates of 47.1 and 45.3 per 1,000 among Canadians aged 18 to 24 and 25 to 44, respectively (*Internal Migration: Overview, 2016/2017 to 2018/2019, 2021*). Considering that some of the internal migrants will migrate again, and that it is likely that migration is less among children and older adults. It is arguable that over the span of 15 years (the time difference in our data), more than half of Canadians will remain in the same CD.

It is worth noting that the variable *Single parenting* is indicative of the family size of single parents. A higher family size, therefore, is also related to more frequent reproduction. The family size of single parents is a particular case of reproduction, though. Parental absence—usually the father—has been proposed as the strongest predictor of a faster LHS (Ellis et al., 2003; Hartman et al., 2018; Webster et al., 2014). In addition to increasing levels of harshness and unpredictability in the child's environment, parental absence could cue children that relationships are not lasting and that some partners do invest less in their offspring. This expectation would later in life result in lower investment in relationships, relatively higher investment in short-term relationships, and lower investment in offspring, creating a cycle of parental absence (Del Giudice et al., 2015; Stearns, 1992; Volk, 2023).

Naive linear models had fewer errors than the PLS-SEM in predicting the outcome variables. This has been a consistent observation in the multiple model iterations in this and in past studies, and it probably indicates that the factors we are using to describe the measures are not in fact functioning as factors. This would be an expected or common result when dealing with secondary data because the measures were not designed to be grouped as factors. On the other hand, both the naive linear model and the PLS model incurred considerably low errors. The RMSE varied between 2.06% and 15.4% of the mean value and between 41.9% and 71.9% of the standard deviation of the variables in *Frequent reproduction*. This indicates that both models’ errors were only a fraction of the mean value and lower than 1 standard deviation of any of the variables. Therefore, both models had errors smaller than the natural variability of the data, which is something remarkable given the reduced sample for the census divisions and the expected noisy characteristics of such a dataset.

A smaller issue regards the discriminant validity between the variables (HTMT). Indeed, the measure used in *Young Children* (i.e., the percentage of children between 0 and 4 years of age) is one of the measures loading into *Frequent reproduction*, only with the 15-year gap difference. The HTMT not meeting criteria was only the case in the bootstrapped confidence interval upper limit, though, and the Fornell-Lacker criterion was within recommended values. Therefore, we decided to keep *Young Children* in the model. Further interpretation of this issue and of the paths between predictors and outcome will be approached in the main discussion. For studies 2, 3, and 4, we decided to use CD data and the model built with CD data as the focus of the subsequent analyses.

## Study 2: Is It a Developmental Phenomenon or Just Statistical Artifacts?

In this study we were interested in testing the hypothesis that the results found were a mere correlational stability of our variables across time. Whether the findings of study 1 likely describe a developmental association or merely statistical artifacts. To achieve that, we reversed the years of predictor and outcome variables. We set the variables of harshness and unpredictability in 2021 to predict reproduction variables in 2006. Since our primary hypothesis is that harsh and unpredictable environments would predict more frequent reproduction in Canadian geographies, we expected that the hypothesis of mere correlational stability would not be supported. Therefore, we hypothesized that the longitudinal model in study 1 will have a better performance and a higher explanatory power than the model with the reversed timeline.

### Method

We used the CD data frame because it had higher predictive power in study 1. The model in this study started with the first iteration of the CD model in the previous study. However, the predictor variables were selected from the 2021 Census, and the outcome variables were selected from the 2006 Census. Similar to the previous study, we established a threshold of a difference of *R*^2^ > .1 to consider that the models are performing differently in their explanatory power. By following the same methods, we attempted to make a fair comparison between the developmental model—with the correct timeline between predictors and outcomes—and the reversed model, with the future predicting variables in the past. We expected that random variations in the data would result in the models being slightly different, but we argue that their performances would be more comparable because we found the same methods.

### Results

**Figure 4.**
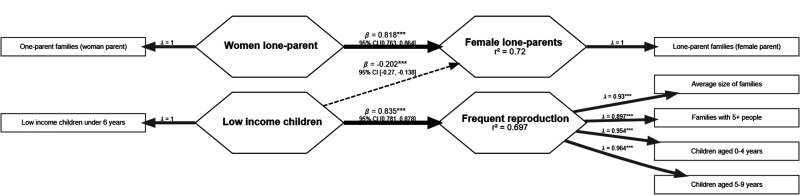
Later harshness and unpredictability are poor predictors of previous measures of early reproduction.

*Note*. Figure created in R using seminr package. Hexagons represent latent variables and rectangles represent items from census. Predictors are formative latent variables using census data in 2006 and outcomes are reflective latent variables using census data in 2021. DA = dissemination areas; CD = census divisions; W: variable's weights; λ: variable's loadings; β: path's beta coefficients; **: *p* ≤ .01, ***: *p* ≤ .001.

Only two single-item predictors and two outcome variables remained in the reversed timeline model. *Low-income children* had a positive path to predict *Frequent reproduction* and a negative one to predict *Female lone-parents* and *Female lone-parents* also had a positive path to predict *Female lone-parents* 15 years in the past (Figure 4). Since the predictors were all single-item variables, formative model assessment does not apply to this model.

The reflective model assessment of *Frequent reproduction* resulted in acceptable measures of factor quality and indicated a cohesive factor. The loadings were *Average size of families* = 0.93, *Families with 5 or more people* = 0.90, *Children aged 0–4 years* = 0.95, and *Children aged 5–9 years* = 0.96. The indicators’ reliability were: *Average size of families* = .87, *Families with 5 or more people* = .80, *Children aged 0–4 years* = .91, *Children aged 5–9 years* = .93; and the factor internal consistency indices were α = .95, ρC = .97, ρA = .94; and reliability was AVE = .88. The 99.5% confidence interval of the HTMT discriminant validity criterion also did not pass 1 with any of the other factors, but it was above .9 with *Low-income children*. The Fornell-Larcker criterion showed a higher square root of the AVE of *Frequent reproduction* than its correlations with the other factors, which indicates discriminant validity.

Figure 4 reports the structural model in study 2. The model assessment indicated no collinearity between the predictors of *Female lone-parents* (VIF = 1.00); all paths were relevant (*β* *≥* |.1|) and statistically significant (*p* ≤ .01) with CI not including 0. The explanatory powers of both *Frequent reproduction* and *Female lone-parents* were moderate (adjusted *R*^2^ = .70 and .72, respectively). The analysis revealed large effect sizes from *Female lone-parents* to *Female lone-parents* (*f*^2^ = 2.40) and from *Low-income children* to *Frequent reproduction* (*f*^2^ = 2.30) and a medium effect size from *Low-income children* to *Female lone-parents* (*f*^2^ = 0.15).

Both out-of-sample metrics of predictive values were lower on the linear model than on the partial least squares model. RMSE LM: *Average size of families* = .080, *Families with 5 or more people* = .420, *Children aged 0–4 years* = .538, *Children aged 5–9 years* = .498, *Lone-parent families (female parent)* = .302; RMSE PLS: *Average size of families* = .084, *Families with 5 or more people* = .471, *Children aged 0–4 years* = .569, *Children aged 5–9 years* = .570, *Lone-parent families (female parent)* = .359; MAE LM: *Average size of families* = .062, *Families with 5 or more people* = .317, *Children aged 0–4 years* = .411, *Children aged 5–9 years* = .393, *Lone-parent families (female parent)* = .232; and MAE PLS model: *Average size of families* = .066, *Families with 5 or more people* = .370, *Children aged 0–4 years* = .427, *Children aged 5–9 years* = .448, *Lone-parent families (female parent)* = .291. This indicates that a naive LM performs better at predicting *Female lone-parents* and *Frequent reproduction* regardless of the data distribution.

One predictor that was excluded from the model is worth noting. The percentage of children aged 0 to 4 years was removed because it was highly collinear with other predictors (VIF = 21.0). This finding was dissimilar to the models in study 1. Several of the predictors were removed because they were not relevant (*β* ≤ .1) and/or not statistically significant (*p* ≥ .01 and/or CI including 0). They were *Low schooling, Male lone-parents*, *Indigenous peoples*, *Precarious labor*, and *Visible minorities*. The eighth iteration of the model in *SM_Study2* reports these. *SM_Study2* provides a full report on the iterations to reach the model.

### Discussion

The reversed model had only two manifest variables predicting two factors: *Female lone-parents* (a single-item factor) and *Frequent reproduction* (a factor composed of 4 variables). It is remarkable that only two predictors were able to predict 72% and 70% of the variances of these outcomes. A further examination of these predictive pathways, however, may facilitate a more comprehensive interpretation. The path on the top of Figure 4 describes the percentage of *Female lone-parents* in 2021, predicting the percentage of *Female lone-parents* in 2006. This is a variable predicting itself, which means we’re merely observing a geographical association of the variable over time. Therefore, this path offers little to no relevant information.

The outcome *Female lone-parents* is also being predicted by *Low-income children* with a very significant path (*p* ≤ .001), but with a lower coefficient. This association was similar to the one found in the CD model in study 1, in which the prevalence of low-income children predicted the family size of one-parent families. Surprisingly and differently from the association in study 1, the association in this study was negative, meaning that a higher percentage of *Low-income children* in 2021 is associated with a lower percentage of *Female lone-parents* in 2006. This contradicts common PAT literature (Hartman et al., 2018; Volk, 2025). We must highlight, however, that this timeline is reversed. Even though there are some suggestions of a repetitive pattern in LHS across generations (Del Giudice et al., 2015), LHT-P literature offers little support that faster strategists will have children that will live in harsher environments.

*Low-income children* also predicts *Frequent reproduction* remarkably well. When we take into account that this is a temporally flipped model, this results in an argument that CD with a higher family size and a higher percentage of children will experience a higher percentage of children living in low-income households 15 years later. Finally, several of the predictors were removed from the model because they were not relevant and/or not significant. This potentially indicates that reversing the timeline does not result in an acceptable model (i.e., iterations of the model do not improve the paths or the explanatory power, and the model does not stabilize with such variables). The failure to reach an acceptable model with a reverse timeline supports our hypothesis that this is a developmental phenomenon and argues against the alternative hypothesis that the findings are merely statistical artifacts.

## Study 3: Is There a Sensitive Period to Experience Harsh and Unpredictable Environments?

Here we are interested in further testing the developmental hypothesis that children who experience harshness and unpredictability are likely to have reproduced 15 years later instead of the alternative hypotheses that the model in study 1 is merely describing a geographical correlation or a statistical artifact. PAT literature points to the first 5 or 7 years of life as the most sensitive period in which exposure to harsh and unpredictable environments can shape reproductive patterns (Ellis et al., 2003; Simpson et al., 2012; Webster et al., 2014; Xu et al., 2018). We hypothesized that models using quantile subsamples of Canadian CD with the highest percentage of children will have better performance at predicting reproductive patterns than CD with a lower percentage of children.

### Method

We subsampled the data into quantiles: (1) highest percentage of children aged 0 to 4 years; (2) lowest percentage of children aged 0 to 4 years; (3) highest percentage of children aged 5 to 9 years; (4) lowest percentage of children aged 5 to 9 years. The inclusion of such year gaps was intended to cover the 0- to 7-year range in which children would be most sensitive to their environment to adjust their LHS. This choice was also motivated by the fact that fertility has been globally declining (Roser, 2014) and the average age of the parents at the birth of the child has been increasing in Canada (Provencher & Galbraith, 2024). An older year range for children would, therefore, allow for a more probable association to adults at reproductive age 15 years later.

The CD sample was a subsample of tertiles of the data, given that it is composed of only 240 cases, while quartiles were used in the DA sample because it is a much bigger data frame. The final subsamples were composed of 80 cases for the CD tertiles and 9871 for the DA quartiles. We repeated the models reached in study 1 with the CD and DA subsamples. For brevity and simplicity, only the results observed with the CD sample—the model with better performance in study 1—will be reported in this manuscript. The full report can be found in *SM_Study3.* Because we aimed at having the most comparable model possible between the models using subsamples in this study and the model in study 1, we did not assess the measurement model's quality and focused solely on the structural model reports in this comparison.

### Results

Table 4 reports the collinearity values, coefficients, effect sizes, and explanatory power of CD tertiles with the highest and lowest percentage of children aged 0 to 4 years and Table 5 reports the same metrics with the highest and lowest percentage of children aged 5 to 9 years. Considering our established criteria of a *β* ≥ |.1| for assuming a path relevance and of an adjusted *R^2^ difference* ≥ .1 as an indicator of a different explanatory power, the models using the subsample with the highest percentage of children in both age groups better explained the variance of S*ingle parenting*. Also considering the same criteria, the models’ performance at predicting *Frequent reproduction* were similar.

**Table 4.** Structural Model Assessment of the Tertiles of Children Aged 0 to 4 Years in Study 3.

	Highest Percent of Children Aged 0–4 Years			Lowest Percent of Children Aged 0–4 Years		
Predictors	Frequent Reproduction			Frequent Reproduction		
	VIF	Paths	f^2^	VIF	Paths	f^2^
Unemployed	1.00	− 0.51***	0.52	1.03	− 0.45***	0.47
Young children	1.00	0.48***	0.36	1.03	0.56***	0.75
Adjusted R^2^	.51			.59		
**Predictors**	**Single parenting**			**Single parenting**		
Low-income children	2.06	0.48***	0.28	1.64	0.35***	0.13
High rents	1.83	− 0.27**	0.09	1.59	− 0.49***	0.26
Unemployed	1.87	− 0.21*	0.06	1.23	− 0.39***	0.21
Indigenous	2.99	0.48***	0.19	1.25	0.13	0.03
Young children	1.57	0.13	0.03	1.12	0.40***	0.25
Adjusted R^2^	.58			.39		

*Note*. VIF: collinearity assessment. Bootstrapped paths reported. *: *p* ≤ .05, **: p ≤ .01, ***: p ≤ *.001*.

**Table 5.** Structural Model Assessment of the Tertiles of Children Aged 5 to 9 Years in Study 3.

	Highest Percent of Children Aged 5–9 Years			Lowest Percent of Children Aged 5–9 Years		
Predictors	Frequent Reproduction			Frequent Reproduction		
	VIF	Paths	f^2^	VIF	Paths	f^2^
Unemployed	1.03	− 0.54***	0.68	1.07	− 0.37***	0.38
Young children	1.03	0.46***	0.41	1.07	0.64***	1.12
Adjusted R^2^	.59			.65		
**Predictors**	**Single parenting**			**Single parenting**		
Low-income children	1.62	0.50***	0.37	1.72	0.35**	0.11
High rents	1.47	− 0.20*	0.06	1.68	− 0.47***	0.22
Unemployed	1.57	− 0.25*	0.10	1.23	− 0.37***	0.19
Indigenous	1.99	0.47***	0.27	1.15	0.12	0.02
Young children	1.21	0.18*	0.07	1.16	0.48***	0.33
Adjusted R^2^	.56			.37		

*Note*. VIF: collinearity assessment. Bootstrapped paths reported. *: *p* ≤ .05, **: p ≤ .01, ***: p ≤ *.001*.

Interestingly, the percentage of Indigenous people was a statistically significant predictor and had a higher and positive coefficient in the tertile with a higher percentage of children in comparison to the tertile with the lowest percentage. This may indicate an interaction between these variables, but testing this hypothesis was beyond the scope of this study. Counterintuitively, *Young children* had a stronger influence on both *Single parenting* and *Frequent reproduction* in the tertiles with the lowest percentage of children. This is counterintuitive because this is the variable we used to subsample the data in tertiles (i.e., highest and lowest percentage of children aged 0–4 years). This may be due to greater variability in the subsample with the lowest percentage of children. A further inspection of means and standard deviations of the tertiles indicated that this greater variability is unlikely to be the case (lowest tertile M = 4.07, SD = 0.32; highest tertile M = 6.05, SD = 0.61). This could also be due to the marginal effects in the lowest tertile. Since these lowest tertiles already have a reduced number of children, the comparative effect of adding a small proportion of children can have a greater impact on the model. Future studies could further explore these associations.

### Discussion

Overall, PAT claims were supported when the model was predicting *Single Parenting*, but not when predicting *Frequent reproduction.* Economic conditions, particularly *Unemployed* and *High rents* in 2006, consistently and negatively predicted *Frequent reproduction* and *Single parenting* in 2021. This finding is consistent with the findings in study 1, and they contradict the theory that exposure to harshness and unpredictability accelerates reproduction. Notably, *Unemployed* negative effect was mixed in areas with higher and lower percentages of children depending on whether it was predicting *Single Parenting* or *Frequent reproduction*. The negative association was greater when predicting *Frequent reproduction* in the tertile with the highest percentage of children and when predicting *Single Parenting* in the tertile with the lowest percentage of children. Conversely, the presence of *Young children* had a larger positive association in areas with fewer children, suggesting some sensitivity in demographic patterns to even slight increases in child population.

*Single parenting* predictors varied distinctly between tertiles. *Low-income children* and *Indigenous* people were strongly associated with higher single-parent households, particularly in areas with a higher percentage of children. In the model using CD, *High rents* consistently predicted smaller *Single parenting* and the association was more robust in areas with fewer young children.

## Study 4: Do Indigenous People and Visible Minorities Face Different Circumstances?

Next, we tested whether the percentage of *Indigenous people* or *Visible minorities* would be particularly relevant predictors of reproduction indicators. Indigenous peoples and visible minorities have a long history of facing harsher circumstances than the general population (*Key Health Inequalities in Canada*, 201*8*; Prather et al., 2016). These harsher circumstances may be due to the history of colonization, racism, cultural and other forms of oppression, and discrimination (Isumonah, 2024; Phillips-Beck et al., 2020). These factors indicative of harsher circumstances may not fully be captured in the usual measures of harshness and unpredictability in LHT-P. Therefore, in addition to using the percentage of Indigenous people and visible minorities as predictors of reproduction indicators (study 1), here we hypothesized that models using a quantile subsample of the highest percentage of Indigenous people and visible minorities in Canadian DA and CD would perform better at predicting *Frequent reproduction* than models using the lowest quantiles.

### Method

The method in this study followed the same procedure as the methods in study 3. However, in this study the subsamples using CD data were selected by the tertiles with the highest and lowest percentage of visible minorities, and the subsamples using DA data were selected by the quartiles with the highest and lowest percentage of Indigenous people. This choice aimed at avoiding subsampling a dataset using a variable that was already a significant predictor in that model. In other words, since *Indigenous* people was already a significant predictor in the CD model and *visible minorities* was already a significant predictor in the DA model, we decided to only select subsamples using the nonsignificant variable. This choice aimed to reduce the likelihood that marginal effects, hypothesized in study 3, would have an effect on the quantiles in this study. We focused our analyses on the CD model (the one performing better in study 1), but we will briefly report some of the statistics in the DA model. The full report can be found in *SM_Study4*.

### Results

Table 6 reports the structural model measurements of CD tertiles. The models performed mostly similarly based on our criteria for statistical significance (*p* ≥ .01 and/or CI including 0), relevance (*β* ≤ .1), and explanatory power (R^2^ difference > .1). The only two exceptions were *High rents*, which had a smaller negative association with *Single parenting* in the tertile with the lowest percentage of *Visible minorities*, and the *Unemployed* prediction of *Single parenting*, which was no longer significant in the lowest tertile. The models using the DA quartiles did not show any relevant difference between the areas with the highest and lowest percentage of visible minorities (see *SM_Table_1* in *SM_Study4*).

**Table 6.** Structural Model Assessment of the Tertiles of Visible Minorities in Study 4.

	Highest Percent of Visible Minorities			Lowest Percent of Visible Minorities		
Predictors	Frequent Reproduction			Frequent Reproduction		
	VIF	Paths	f^2^	VIF	Paths	f^2^
Unemployed	1.08	− 0.41***	0.69	1.2	− 0.42***	0.93
Young children	1.08	0.69***	2.07	1.2	0.66***	2.27
Adjusted R^2^	.79			.84		
**Predictors**	**Single parenting**			**Single parenting**		
Low-income children	1.87	0.42***	0.25	1.41	0.31***	0.23
High rents	1.98	− 0.35***	0.16	1.24	− 0.11	0.03
Unemployed	1.70	− 0.18*	0.05	1.44	− 0.13	0.03
Indigenous	1.79	0.20*	0.06	1.97	0.22**	0.07
Young children	1.57	0.60***	0.63	1.97	0.52***	0.45
Adjusted R^2^	.62			.66		

*Note.* VIF: collinearity assessment. Bootstrapped paths reported. *: *p* ≤ .05, **: p ≤ .01, ***: p ≤ *.001.*

### Discussion

Contrary to our prediction, selecting geographies with the highest and lowest percentages of Indigenous people and visible minorities did not affect the models’ performances. The percentage of *Visible minorities* did not substantially affect the model performance in Canadian CD, and the percentage of *Indigenous people* did not affect the model performance in Canadian DA. This may be explained by the observations that these variables were not significant in the first models (using the whole sample); therefore, they would not substantially have an effect on their subsamples.

It is interesting and counterintuitive, though, that *Indigenous people* and *Visible minorities* were only significant or relevant predictors in the CD and DA samples, respectively. Since these samples are only different geographical organizations of the same population in the same year, one could expect the same variables to be significant predictors in both models. One explanation for that could be that *Indigenous people* and *Visible minorities* are significant predictors of different variables in the two models. *Indigenous people* is only a significant and relevant predictor of *Single parenting* in the CD model, whereas *Visible minorities* is only a significant predictor of *Frequent reproduction* in the DA model. This would not explain why *Visible minorities* was not a significant predictor of *Frequent reproduction* in the CD model, though.

Another explanation could relate to migration. DA are small geographic areas and susceptible to high migration. Even though CD are the most stable geographic unit (*Dictionary, Census of Population, 2021*, 202*3*), they are still susceptible to migration in a time span of 15 years. Canada has also observed high immigration from other countries in recent years (Statistics Canada, 2022), so it is likely that a considerable proportion of people answering the census in 2021 were not even in the country in 2006.

A final explanation could be related to the skewness of the data. As with many variables used in this manuscript, the percentages of *Indigenous people* and *Visible minorities* were considerably low (*Indigenous people* in CD sample: M = 10.9, SD = 16.0; and *Visible minorities* in CD sample: M = 3.09, SD = 6.05). If one of these variables exhibited greater variance in one of the geographic divisions but not in the other geographic division, it could disproportionately influence the model and become a significant or relevant predictor. Indeed, the percentage of *Indigenous people* and of *Visible minorities* was many times more than 50% of the population in the DA data frame, sometimes being the entire population of that area. The same does not occur in the CD sample. In sum, the hypothesis that subsampling *Indigenous people* or *Visible minorities* would increase model performance was not supported. Future studies could further explore how marginalized or discriminated populations face harsher and more unpredictable circumstances and how that can relate to PAT assumptions.

## General Discussion

The results point to the viability of using census data, a longitudinal design, and an exploratory multivariate analytical approach to test common PAT assumptions. The models built with data from both geographic divisions could explain remarkable variance of reproduction patterns. Namely, the CD, which is the bigger and more stable geographic division (*Dictionary, Census of Population, 2021*, 202*3*), was able to explain 81% of the variance of indicators of larger family sizes (named here *Frequent reproduction*) and 64% of the variance of the family size of one-parent families in 2021 using predictors in 2006. The DA, which is a smaller geographic division and expected to be a less stable geographic division over time, explained 49% of the variance of similar variables in the same time frame.

These point to both geographic divisions being predictive of reproductive patterns in the Canadian population. The DA geographic division allows for more granular, although noisier, prediction, and the CD division allows for greater population and more precise prediction. For the aims in this article, the CD was the best-performing one and is the focus of this study. In general, these findings support that the use of such data and methodologies can effectively project future reproduction trends among Canadians, which can be a useful tool for policy development and various governmental initiatives.

The effect sizes that were found are high in comparison to what is typically reported in psychology studies. Studies in psychology are usually able to explain around 40% of variance (i.e., *R*^2^ = .4), and the models reported in study 1 are able to explain between 49% and 81% of the outcome variables.

These results could be due to (1) the statistical power that census data offer; (2) the relatively exploratory analytical approach that we used; and (3) the PLS-SEM algorithm to calculate such effects. The census is the best description of a population. Even though we used percentages or mean values—which inherently reduces some of the variance—of Canadian CD or DA, we still analyzed data collected from millions of people. This statistical power could partly explain the remarkably high effects that have been found.

The first motivator to select variables was the variables present on the Canadian census (Statistics Canada, 2024) that were similar to usual measures of harshness, unpredictability, and reproduction in PAT research. After that, variables were removed from the model or regrouped in different factors for statistical reasons. This relatively exploratory analytical approach could also result in inflated findings. Finally, PLS-SEM algorithm has been criticized for how it calculates statistical significance and for inflating some of its reported values (Rönkkö et al., 2015). This was part of the reason why we adopted criteria that were stricter than what is conventional in research in psychology (e.g., *p* < .1, *R*^2^ > .2).

The findings also mostly support the longitudinal and developmental aspects of PAT (Ellis et al., 2003; Simpson et al., 2012; Webster et al., 2014; Xu et al., 2018). In study 1, both models had the percentage of children (aged 0–4 in the CD sample and 0–9 in the DA sample) as the highest predictor of reproductive patterns 15 years later. The percentage of children under 6 years old in low-income families was also a significant predictor of the family size of one-parent families in the CD model. When we reversed the timeline between predictor and outcomes, a lot of the variables emerged as nonsignificant predictors, consistent with the idea that this is a developmental phenomenon. Several of the variables were not significant or not relevant in the reversed timeline model, which could mean that their prediction of reproduction was noisier (i.e., resulting in greater error). Reversing the timeline did not result in a prediction as accurate as the correct timeline, which, again, supports the argument of a developmental phenomenon instead of the hypothesis of geographical association.

Subsampling quantiles of the percentage of children did not result in consistently greater performance of the quantiles with a higher percentage of children. The explanatory power was greater in predicting *Single parenting*, but not in predicting *Frequent reproduction*. However, this lower prediction of *Frequent reproduction* is not surprising because this variable was being predicted by the percentage of young children and by *Unemployed*. By selecting the tertiles of highest and lowest young children, we are essentially reducing its variance in both tertiles and leaving *Unemployed* as the only predictor fully varying in the model. This did not happen when predicting *Single parenting* because there were more predictors. Another issue is that the variable percentage of *Children aged 0–4 years* was a predictor, the variable used for the quantile subsampling*,* and an outcome of *Frequent reproduction*—although it was measured 15 years later. This allows for alternative interpretations and obscures the phenomenon. This issue is not present in the prediction of *Single parenting.*

The PAT claim that environmental harshness and unpredictability (Ellis et al., 2009; Stearns, 1992) shape reproductive patterns was not strongly supported. The significant indicators in the CD sample—*Unemployed* and *High rents—*were actually negatively associated with *Frequent reproduction* and *Single parenting* in the CD sample. A similar pattern of results happened in the DA sample, in which *Lack of resources* and *Divorced or Widowed* were negatively associated with *Frequent reproduction.* The percentages of divorced and widowed people were assumed to be indicative of parental transition, which is the biggest predictor of early and frequent reproduction in PAT literature. The negative association in the Canadian population works against this hypothesis. Median family income was positively associated with *Frequent reproduction* (i.e., wealthier CD in 2006 were associated with a higher percentage of children in 2021)*.* These results support the argument that modern environments, particularly the ones in high-income countries, may not be reflective of the harshness and unpredictability encountered in our environment of evolutionary adaptedness and that would cue different reproductive patterns (Nolin & Ziker, 2016; Volk, 2023). This claim is supported by a study that we conducted with data from Brazil—a lower-income country with considerable income inequality—using a similar method and similar analytical approach (Koehler & Rutherford, 2026). In this study, harshness indicators were predictive of and positively associated with early reproduction. In this same report, we tested whether a model similar to the one built with data from Brazil would predict reproductive outcomes in US districts. Some of the variables were statistically significant predictors of reproductive data, but contrary to what would be expected according to PAT assumptions. For example, districts with higher percentages of households lacking complete plumbing facilities, higher percentages of divorced people, and higher percentages of visible minorities negatively predicted the percentages of children between 0 and 4 years old. The results found in the US contrast withthe findings in Brazil and are similar to the results found here. These findings can further support the argument that the levels of harshness and unpredictability in modern environments—particularly in high-income countries—are different from those found in our evolutionary history.

There are, of course, explanations alternative to PAT that can help explain this phenomenon. Fertility has been dropping, and the time of first pregnancy is also being delayed worldwide (Roser, 2014), including in Canada (Provencher & Galbraith, 2024). These are thought to be the result of women having more access to education, healthcare, and employment (Behrman & Gonalons-Pons, 2020; Olowolafe et al., 2025) and of the reduction in child mortality (Roser, 2014). While these may be reflective of a more standardized, stable, and less harsh environment, there are other explanations, such as lack of institutional support for effective reproductive plan and control (South & Crowder, 2010; Wodtke, 2013), local cultures and values (Wilson, 1987; Wodtke, 2013), and local contagion (i.e., observing others having children or having children at a younger age could cue others in the community to also have children; South & Crowder, 2010).

Even within the scope of LHT-P, there are alternative explanations not considered in these analyses. The influence of genes, for instance, was not tested. Gene-environment interactions are the basis for evolutionary research (Stearns, 1992), and genetics should be playing a role in the phenomenon explored in this study. Genes influence the time of puberty (Del Giudice et al., 2015), and the same genes that select for or shape certain environments can also influence reproductive behavior (Belsky et al., 1991; Volk, 2025). In any case, these alternative explanations seem to agree that fertility is higher in places with harsher, resource-lacking conditions. Therefore, the results pointing to geographies with these harsher environments experiencing less *Frequent reproduction* and *Single parenting* are surprising, and future research could focus on understanding such dynamics.

The percentage of *Indigenous people* was a significant and positively associated predictor of *Single parenting* in the CD model, and the percentage of *Visible minorities* was a significant and positively associated predictor of *Frequent reproduction* in the DA model. These being significant predictors in addition to the indicators of a harsher or more unpredictable life included in the model can be interpreted as evidence that there are particularities of these communities that are not being measured by the model.

The long history of colonization, structural inequities (Goghari & Kassan, 2022) and the confinement of Indigenous people in “reservations” (Neu & Graham, 2006; Romaniuk, 2008) can create specific harsh and unpredictable environments that are not usually measured in PAT literature. Indigenous people and visible minorities suffer more marginalization and discrimination (Prather et al., 2016), experience or live in communities with higher levels of violence and crime (Griskevicius et al., 2011; Williams et al., 2022; M. Wilson & Daly, 1997), experience cultural differences from non-Indigenous or Caucasian ethnicities (Trovato & Burch, 1980), or experience differences in access to institutional support such as health care, education, or child care (Roser, 2014; Wilson, 1987; Wodtke, 2013). These are factors historically neglected by Western society that could be influencing the reproductive behavior of both Indigenous people and visible minorities in Canada. Future studies could explore how LHT-P can interact with the environments of these populations.

### Caveats

There are many considerations and limitations to this study. The first is that this study uses geography-level data; therefore, conclusions about individuals are remarkably limited. Considering PAT and other sources, we draw some conclusions about individual circumstances and behaviors, but it is possible that associations at a population level do not exist at the level of the individual. For example, Canada has been experiencing a considerable amount of immigration (Statistics Canada, 2022), which is commonly associated with employment or education. Immigrants in Canada also have more children than their Canadian-born counterparts (Bélanger et al., 2006). Therefore, it may be likely that the geographies accepting more immigrants are geographies that tend to have lower unemployment rates and more children, but these would not be the same individuals or the same households.

The conclusions drawn here rely on an assumption that most of the population of a given geography will remain on the same geography 15 years later. This is not a guarantee, especially in a country with a high level of immigration (Statistics Canada, 2022) or particularly in smaller geographic units such as DA. Research conducted with a cohort of more than 800,000 people from the Canadian Community Health Survey identified that 54% of them moved within the past 10 years. However, around 37% of them are likely to have moved within the same CD (Mah et al., 2025), resulting in around 34% of the population moving to a different CD in a 10-year period. Arguing that the results from this survey apply to the entire country population and to the findings in this manuscript is a considerable extrapolation, though.

Another limitation of this study is its exploratory analytical approach. The choice of variables in this study was based on a largely used theory (PAT) and some of the tests and comparisons conducted were aimed at assessing the likelihood of statistical artifacts or alternative explanations. However, we started the first iterations of the models with 40 variables and selected or removed variables for statistical reasons. In addition, the analyses were all conducted with the same data from two time points (2006 and 2021). Future research should aim at confirming these findings to allow for more generalizable, reliable or even causal conclusions.

Finally, as mentioned earlier, LHT-P and PAT have been going on through serious criticisms (Nettle & Frankenhuis, 2020; Sear, 2020; Volk, 2025). These criticisms argue for the need of reconsideration of core aspects of the theories and toward a re-approximation to its origin in biology (Frankenhuis & Nettle, 2020, 2020; Stearns & Rodrigues, 2020; Volk, 2025). This significantly limits the inferences and conclusions that can be drawn from this study, but it also highlights its importance. More research using data descriptive of entire populations and aiming at specific outcomes can help LHT-P and PAT to refine its assumptions and generate more accurate and formal predictions.

### Conclusion

Using Canadian census data, we showed that indicators of harshness and of the proportion of children and visible minorities can predict frequent reproduction and family sizes 15 years later. We also showed that using data from CD results in a more accurate model than data from DA. The proportion Indigenous people and Visible minorities in Canada are significant and relevant predictors of such reproductive outcomes, highlighting the importance of understanding their structural and cultural characteristics to better understand the ecological and social factors shaping reproductive strategies and outcomes. These findings help inform future research that can make use of more confirmatory approaches to confirm these results (Hair et al., 2022) and to make use of more formal modeling (Frankenhuis & Nettle, 2020; Nettle & Frankenhuis, 2020). These results and future results can also inform an array of public policies, especially those aiming at dealing with early pregnancy and family planning of Indigenous peoples and visible minorities.

## Supplemental Material

sj-docx-1-evp-10.1177_14747049261432881 - Supplemental material for Childhood Demographics and Socioeconomic Conditions Predict Reproduction 15 Years Later Supplemental material, sj-docx-1-evp-10.1177_14747049261432881 for Childhood Demographics and Socioeconomic Conditions Predict Reproduction 15 Years Later by Vinícius Betzel Koehler and M.D. Rutherford in Evolutionary Psychology

sj-zip-2-evp-10.1177_14747049261432881 - Supplemental material for Childhood Demographics and Socioeconomic Conditions Predict Reproduction 15 Years Later Supplemental material, sj-zip-2-evp-10.1177_14747049261432881 for Childhood Demographics and Socioeconomic Conditions Predict Reproduction 15 Years Later by Vinícius Betzel Koehler and M.D. Rutherford in Evolutionary Psychology

## References

[bibr1-14747049261432881] Albaladejo-Robles G. Böhm M. Newbold T. (2023). Species life-history strategies affect population responses to temperature and land-cover changes. Global Change Biology, 29(1), 97–109. 10.1111/gcb.16454 36250232 PMC10092366

[bibr2-14747049261432881] Behrman J. Gonalons-Pons P. (2020). Women’s employment and fertility in a global perspective (1960–2015). Demographic Research, 43(25), 707–744. 10.4054/DemRes.2020.43.25 34305448 PMC8295801

[bibr3-14747049261432881] Bélanger A. Martel L. Gilbert S. Berthelot J.-M. (2006). Report on the Demographic Situation in Canada, 2002. Statistics Canada. http://www.statcan.ca

[bibr4-14747049261432881] Belsky J. Schlomer G. L. Ellis B. J. (2012). Beyond cumulative risk: Distinguishing harshness and unpredictability as determinants of parenting and early life history strategy. Developmental Psychology, 48(3), 662–673. 10.1037/a0024454 21744948

[bibr5-14747049261432881] Belsky J. Steinberg L. Draper P. (1991). Childhood experience, interpersonal development, and reproductive strategy: An evolutionary theory of socialization. Child Development, 62(4), 647–670. 10.1111/j.1467-8624.1991.tb01558.x 1935336

[bibr6-14747049261432881] Buss D. M. (2024). Evolutionary psychology: The new science of the mind (7th edition). Routledge.

[bibr7-14747049261432881] Copping L. (2017). Census data. In Shackelford T. K. Weekes-Shackelford V. A. (Eds.), Encyclopedia of evolutionary psychological science (pp. 1–5). Springer International Publishing. 10.1007/978-3-319-16999-6_1852-1

[bibr8-14747049261432881] Copping L. T. Campbell A. (2015). The environment and life history strategies: Neighborhood and individual-level models. Evolution and Human Behavior, 36(3), 182–190. 10.1016/j.evolhumbehav.2014.10.005

[bibr9-14747049261432881] Copping L. T. Campbell A. Muncer S. (2013). Violence, teenage pregnancy, and life history: Ecological factors and their impact on strategy-driven behavior. Human Nature (Hawthorne, N.Y.), 24(2), 137–157. 10.1007/s12110-013-9163-2 23653372

[bibr10-14747049261432881] Del Giudice M. (2009). Sex, attachment, and the development of reproductive strategies. Behavioral and Brain Sciences, 32(1), 1–21. 10.1017/S0140525X09000016 19210806

[bibr11-14747049261432881] Del Giudice M. Belsky J. (2010). The development of life history strategies: Toward a multi-stage theory. In Buss D. M. Hawley P. H. (Eds.), The evolution of personality and individual differences (pp. 154–176). Oxford University Press. 10.1093/acprof:oso/9780195372090.003.0006

[bibr12-14747049261432881] Del Giudice M. Kaplan H. S. Gangestad S. W. (2015). Life history theory and evolutionary psychology. In Buss D. M. (Ed.), The handbook of evolutionary psychology (pp. 68–95). John Wiley & Sons, Inc. 10.1002/9780470939376.ch2

[bibr13-14747049261432881] Dictionary, Census of Population, 2021. (2023). Statistics Canada = Statistique Canada.

[bibr14-14747049261432881] Dinh T. Haselton M. G. Gangestad S. W. (2022). Fast” women? The effects of childhood environments on women’s developmental timing, mating strategies, and reproductive outcomes. Evolution and Human Behavior, 43(2), 133–146. 10.1016/j.evolhumbehav.2021.12.001

[bibr15-14747049261432881] Ellis B. J. Bates J. E. Dodge K. A. Fergusson D. M. John Horwood L. Pettit G. S. Woodward L. (2003). Does father absence place daughters at special risk for early sexual activity and teenage pregnancy? Child Development, 74(3), 801–821. 10.1111/1467-8624.00569 12795391 PMC2764264

[bibr16-14747049261432881] Ellis B. J. Figueredo A. J. Brumbach B. H. Schlomer G. L. (2009). Fundamental dimensions of environmental risk: The impact of harsh versus unpredictable environments on the evolution and development of life history strategies. Human Nature, 20(2), 204–268. 10.1007/s12110-009-9063-7 25526958

[bibr17-14747049261432881] Frankenhuis W. E. Nettle D. (2020). Current debates in human life history research. Evolution and Human Behavior, 41(6), 469–473. 10.1016/j.evolhumbehav.2020.09.005

[bibr18-14747049261432881] Goghari V. M. Kassan A. (2022). Building a socially and culturally responsive psychology / engendrer une psychologie plus réceptive sur le plan social et culturel. Canadian Psychology / Psychologie Canadienne, 63(4), 467–470. 10.1037/cap0000351

[bibr19-14747049261432881] Griskevicius V. Delton A. W. Robertson T. E. Tybur J. M. (2011). Environmental contingency in life history strategies: The influence of mortality and socioeconomic status on reproductive timing. Journal of Personality and Social Psychology, 100(2), 241–254. 10.1037/a0021082 20873933 PMC3556268

[bibr20-14747049261432881] Hair J. F. Hult G. T. M. Ringle C. M. Sarstedt M. (2022). A primer on partial least squares structural equation modeling (PLS-SEM) (3rd edition). Sage.

[bibr21-14747049261432881] Hair J. F. Hult G. T. M. Ringle C. M. Sarstedt M. Danks N. P. Ray S. (2021). Partial Least Squares Structural Equation Modeling (PLS-SEM) using R: A workbook. Springer International Publishing. 10.1007/978-3-030-80519-7

[bibr22-14747049261432881] Hair J. F. Risher J. J. Sarstedt M. Ringle C. M. (2019). When to use and how to report the results of PLS-SEM. European Business Review, 31(1), 2–24. 10.1108/EBR-11-2018-0203

[bibr23-14747049261432881] Hartman S. Sung S. Simpson J. A. Schlomer G. L. Belsky J. (2018). Decomposing environmental unpredictability in forecasting adolescent and young adult development: A two-sample study. Development and Psychopathology, 30(4), 1321–1332. 10.1017/S0954579417001729 29212568

[bibr24-14747049261432881] Henry F. Ginzberg E. , Urban Alliance on Race Relations, & Social Planning Council of Metropolitan Toronto. (1985). *Who gets the work? : A test of racial discrimination in employment.* Urban Alliance on Race Relations and Social Planning Council of Metropolitan Toronto.

[bibr25-14747049261432881] Honouring the truth, reconciling for the future: Summary of the final report of the Truth and Reconcilliation Commission of Canada. (2015). Truth and Reconcilliation Commission of Canada.

[bibr26-14747049261432881] Husbands W. Lawson D. O. Etowa E. B. Mbuagbaw L. Baidoobonso S. Tharao W. Yaya S. Nelson L. E. Aden M. Etowa J. (2022). Black Canadians’ exposure to everyday racism: Implications for health system access and health promotion among urban black communities. Journal of Urban Health, 99(5), 829–841. 10.1007/s11524-022-00676-w 36066788 PMC9447939

[bibr27-14747049261432881] Internal migration: Overview, 2016/2017 to 2018/2019. (2021). https://www150.statcan.gc.ca/n1/pub/91-209-x/2021001/article/00001-eng.htm

[bibr28-14747049261432881] Intungane D. Long J. Gateri H. Dhungel R. (2024). Employment barriers for racialized immigrants: A review of economic and social integration support and gaps in Edmonton, Alberta. Genealogy, 8(2), 40. 10.3390/genealogy8020040

[bibr29-14747049261432881] Isumonah K. G. (2024). An examination of food insecurity among Canadian Aboriginal people. Journal of Global Health Economics and Policy, 4, Article e2024009. 10.52872/001c.126467

[bibr30-14747049261432881] Johnston M. (2017). Secondary data analysis: A method of which the time has Come. Qualitative and Quantitative Methods in Libraries, 3(3), 619–626.

[bibr31-14747049261432881] Kelly Y. Zilanawala A. Sacker A. Hiatt R. Viner R. (2017). Early puberty in 11-year-old girls: Millennium cohort study findings. Archives of Disease in Childhood, 102(3), 232–237. 10.1136/archdischild-2016-310475 27672135 PMC5339561

[bibr32-14747049261432881] Key health inequalities in Canada: A national portrait : executive summary. (2018). Public Health Agency of Canada = Agence de la santé publique du Canada.

[bibr71-14747049261432881] Koehler V. B. Rutherford M. D. (2026). Harshness predicts reproduction in Brazilian municipalities and US counties: a life history theory approach. *Evolutionary Psychology.* submitted for publication. 10.1177/14747049261432896 PMC13087386 41983907

[bibr33-14747049261432881] Magnus M. C. Anderson E. L. Howe L. D. Joinson C. J. Penton-Voak I. S. Fraser A. (2018). Childhood psychosocial adversity and female reproductive timing: A cohort study of the ALSPAC mothers. Journal of Epidemiology and Community Health, 72(1), 34–40. 10.1136/jech-2017-209488 29122994 PMC5753025

[bibr34-14747049261432881] Mah S. M. Buajitti E. Pagalan L. Diemert L. M. Tjepkema M. Christidis T. Chiodo S. Siddiqi A. Brook J. R. Chen H. Rosella L. C. (2025). Robust indicators of residential mobility derived from longitudinal Canadian data to examine population health across the life course. Social Indicators Research, 177(3), 937–957. 10.1007/s11205-025-03521-0

[bibr35-14747049261432881] Nau R. (2020). What’s a good value for R-squared? https://people.duke.edu/∼rnau/rsquared.htm

[bibr36-14747049261432881] Nettle D. Frankenhuis W. E. (2020). Life-history theory in psychology and evolutionary biology: One research programme or two? Philosophical Transactions of the Royal Society B: Biological Sciences, 375(1803), 20190490. 10.1098/rstb.2019.0490 PMC7293149 32475337

[bibr37-14747049261432881] Neu D. Graham C. (2006). The birth of a nation: Accounting and Canada’s first nations, 1860–1900. Accounting, Organizations and Society, 31(1), 47–76. 10.1016/j.aos.2004.10.002

[bibr38-14747049261432881] Nolin D. A. Ziker J. P. (2016). Reproductive responses to economic uncertainty: Fertility decline in post-Soviet Ust’-Avam, Siberia. Human Nature, 27(4), 351–371. 10.1007/s12110-016-9267-6 27595735

[bibr39-14747049261432881] Olowolafe T. A. Adebowale A. S. Fagbamigbe A. F. Onwusaka O. C. Aderinto N. Olawade D. B. Wada O. Z. (2025). Decomposing the effect of women’s educational status on fertility across the six geo-political zones in Nigeria: 2003–2018. BMC Women’s Health, 25(1), 107. 10.1186/s12905-025-03636-z 40057721 PMC11889893

[bibr40-14747049261432881] Phillips-Beck W. Eni R. Lavoie J. G. Avery Kinew K. Kyoon Achan G. Katz A. (2020). Confronting racism within the Canadian healthcare system: Systemic exclusion of first nations from quality and consistent care. International Journal of Environmental Research and Public Health, 17(22), 8343. 10.3390/ijerph17228343 33187304 PMC7697016

[bibr41-14747049261432881] Prather C. Fuller T. R. Marshall K. J. Jeffries W. L. (2016). The impact of racism on the sexual and reproductive health of African American women. Journal of Women’s Health, 25(7), 664–671. 10.1089/jwh.2015.5637 PMC4939479 27227533

[bibr42-14747049261432881] Provencher C. Galbraith N. (2024). Fertility in Canada, 1921 to 2022 (Demographic Documents No. 91F0015 M). Statistics Canada.

[bibr43-14747049261432881] Reading C. L. Wien F. (2009). Health inequalities and social determinants of Aboriginal peoples’ health. National Collaborating Centre for Aboriginal Health.

[bibr44-14747049261432881] Richardson G. B. Bates D. Ross A. Liu H. Boutwell B. B. (2024). Is reproductive development adaptively calibrated to early experience? Evidence from a national sample of females. Developmental Psychology, 60(2), 306–321. 10.1037/dev0001681 38190216

[bibr45-14747049261432881] Richardson G. B. Placek C. Srinivas V. Jayakrishna P. Quinlan R. Madhivanan P. (2020). Environmental stress and human life history strategy development in rural and peri-urban South India. Evolution and Human Behavior, 41(3), 244–252. 10.1016/j.evolhumbehav.2020.03.003

[bibr46-14747049261432881] Romaniuk A. (2008). History-based explanatory framework for procreative behaviour of aboriginal people of Canada. Canadian Studies in Population, 35(1), 159. 10.25336/P61K7T

[bibr47-14747049261432881] Rönkkö M. McIntosh C. N. Antonakis J. (2015). On the adoption of partial least squares in psychological research: Caveat emptor. Personality and Individual Differences, 87, 76–84. 10.1016/j.paid.2015.07.019

[bibr48-14747049261432881] Roser M. (2014). The global decline of the fertility rate. Our World in Data.

[bibr49-14747049261432881] Sear R. (2020). Do human ‘life history strategies’ exist? Evolution and Human Behavior, 41(6), 513–526. 10.1016/j.evolhumbehav.2020.09.004

[bibr50-14747049261432881] Simpson J. A. Griskevicius V. Kuo S. I.-C ., Sung S. Collins W. A . (2012). Evolution, stress, and sensitive periods: The influence of unpredictability in early versus late childhood on sex and risky behavior. Developmental Psychology, 48(3), 674–686. 10.1037/a0027293 22329381

[bibr51-14747049261432881] South S. J. Crowder K. (2010). Neighborhood poverty and nonmarital fertility: Spatial and temporal dimensions. Journal of Marriage and Family, 72(1), 89–104. 10.1111/j.1741-3737.2009.00685.x 21373376 PMC3046862

[bibr52-14747049261432881] Statistics Canada. (2017). Aboriginal peoples in Canada: Key results from the 2016 Census. The Daily.

[bibr53-14747049261432881] Statistics Canada. (2022). Immigrants make up the largest share of the population in over 150 years and continue to shape who we are as Canadians. The Daily. https://www150.statcan.gc.ca/n1/daily-quotidien/221026/dq221026a-eng.pdf

[bibr54-14747049261432881] Statistics Canada. (2023). Canada's Indigenous population. https://www.statcan.gc.ca/o1/en/plus/3920-canadas-indigenous-population

[bibr55-14747049261432881] Statistics Canada. (2024). Canadian census analyser [Dataset]. Computing in the Humanities and Social Sciences at the University of Toronto (CHASS). https://datacentre.chass.utoronto.ca/census/

[bibr56-14747049261432881] Stearns S. C. (1992). The evolution of life histories. Oxford University Press.

[bibr57-14747049261432881] Stearns S. C. Allal N. Mace R. (2008). Life history theory and human development. In C. Crawford & D. Krebs (Eds.), Foundations of evolutionary psychology (pp. 47–69). Taylor & Francis Group/Lawrence Erlbaum Associates.

[bibr58-14747049261432881] Stearns S. C. Rodrigues A. M. M. (2020). On the use of “life history theory” in evolutionary psychology. Evolution and Human Behavior, 41(6), 474–485. 10.1016/j.evolhumbehav.2020.02.001

[bibr59-14747049261432881] Stone B. W. G. Dijkstra P. Finley B. K. Fitzpatrick R. Foley M. M. Hayer M. Hofmockel K. S. Koch B. J. Li J. Liu X. J. A. Martinez A. Mau R. L. Marks J. Monsaint-Queeney V. Morrissey E. M. Propster J. Pett-Ridge J. Purcell A. M. Schwartz E. Hungate B. A. (2023). Life history strategies among soil bacteria—dichotomy for few, continuum for many. The ISME Journal, 17(4), 611–619. 10.1038/s41396-022-01354-0 36732614 PMC10030646

[bibr60-14747049261432881] Trovato F. Burch T. K. (1980). Minority group Status and fertility in Canada. Canadian Ethnic Studies = Etudes Ethniques Au Canada, 12(3), 1. Periodicals Archive Online.

[bibr61-14747049261432881] Trzesniewski K. H. Donnellan M. B. Lucas R. E. , & American Psychological Association. (Eds.). (2011). Secondary data analysis: An introduction for psychologists (1st ed). American Psychological Association.

[bibr62-14747049261432881] Volk A. A. (2023). Historical and hunter-gatherer perspectives on fast-slow life history strategies. Evolution and Human Behavior, 44(2), 99–109. 10.1016/j.evolhumbehav.2023.02.006

[bibr63-14747049261432881] Volk A. A. (2025). Pumping the brakes on psychosocial acceleration theory: Revisiting its underlying assumptions. Evolution and Human Behavior, 46(1), 106657. 10.1016/j.evolhumbehav.2025.106657

[bibr64-14747049261432881] Webster G. D. Graber J. A. Gesselman A. N. Crosier B. S. Schember T. O. (2014). A life history theory of father absence and menarche: A meta-analysis. Evolutionary Psychology, 12(2), 147470491401200. 10.1177/147470491401200202 PMC10426907 25299880

[bibr72-14747049261432881] Wells J. C. K. Cole T. J. Cortina–Borja M. Sear R. Leon D. A. Marphatia A. A. Murray J. Wehrmeister F. C. Oliveira P. D. Gonçalves H. Oliveira I. O. Menezes A. M. B. (2019). Low maternal capital predicts life history trade–offs in daughters: Why adverse outcomes cluster in individuals. *Frontiers in Public Health* , 7, 206. https://doi.org/10.3389/fpubh.2019.00206 31417889 10.3389/fpubh.2019.00206 PMC6685417

[bibr65-14747049261432881] Williams M. T. Khanna Roy A. MacIntyre M.-P. Faber S. (2022). The traumatizing impact of racism in Canadians of colour. Current Trauma Reports, 8(2), 17–34. 10.1007/s40719-022-00225-5 35345606 PMC8943361

[bibr66-14747049261432881] Wilson M. Daly M. (1997). Life expectancy, economic inequality, homicide, and reproductive timing in Chicago neighbourhoods. BMJ, 314(7089), 1271–1271. 10.1136/bmj.314.7089.1271 9154035 PMC2126620

[bibr67-14747049261432881] Wilson W. J. (1987). The truly disadvantaged: The inner city, the underclass, and public policy. University of Chicago press.

[bibr68-14747049261432881] Wodtke G. T. (2013). Duration and timing of exposure to neighborhood poverty and the risk of adolescent parenthood. Demography, 50(5), 1765–1788. 10.1007/s13524-013-0219-z 23720166 PMC3882124

[bibr69-14747049261432881] Xu Y. Norton S. Rahman Q. (2018). Early life conditions, reproductive and sexuality-related life history outcomes among human males: A systematic review and meta-analysis. Evolution and Human Behavior, 39(1), 40–51. 10.1016/j.evolhumbehav.2017.08.005

[bibr70-14747049261432881] Young E. S. Frankenhuis W. E. Ellis B. J. (2020). Theory and measurement of environmental unpredictability. Evolution and Human Behavior, 41(6), 550–556. 10.1016/j.evolhumbehav.2020.08.006

