# Supplementary material for: Childhood Demographics and Socioeconomic Conditions Predict Reproduction 15 Years Later
Source: Evol Psychol. 2026 Apr 9;24(1):14747049261432881. doi: 10.1177/14747049261432881 (PMC13070147; doi:10.1177/14747049261432881)
Supplement: sj-docx-1-evp-10.1177_14747049261432881 - Supplemental material for Childhood Demographics and Socioeconomic Conditions Predict Reproduction 15 Years Later [file sj-docx-1-evp-10.1177_14747049261432881.docx]

**Appendices**

## Table 1

*Variables fed into the first models in Study 1.*

| **LHT Concept** | **Usual measure in LHT** | **Factor loaded in 1st iteration** | **Variables** | **Final Model** |
| --- | --- | --- | --- | --- |
| Harshness | SES measures | Income | Median family income | DA |
|  |  |  | Prevalence children 6 years of age or less living with low income before tax | CD |
|  |  | Lack of resources | Occupied private dwellings needing minor repairs | DA |
|  |  |  | Occupied private dwellings needing major repairs | DA |
|  |  |  | Tenant occupied households spending more than 30% on rent | CD; DA |
|  |  |  | Employed labour force 15 years of age and over using public transit |  |
|  |  | Low schooling | Population 25 – 64 with no certificate, diploma or degree |  |
| Unpredictability | Parental transitions | Female lone parent | Female lone parent |  |
|  |  |  | Median of female lone-parent income |  |
|  |  |  | Percentage of female lone-parent income coming from other sources (i.e., neither employment nor government transfers) |  |
|  |  | Male lone parent | Male lone parent |  |

**Table 1** (*Continued*)

| **LHT Concept** | **Usual measure in LHT** | **Factor loaded in 1st iteration** | **Variables** | **Final Model** |
| --- | --- | --- | --- | --- |
|  |  |  | Median of male lone-parent income |  |
|  |  |  | Percentage of male lone-parent income coming from other sources (i.e., neither employment nor government transfers) |  |
|  |  | Separated | Divorced | DA |
|  |  |  | Widowed | DA |
|  |  |  | Separated, but still legally married |  |
|  | Parental occupation transitions | Precariously labour | Unemployment rate of population 25 years and over | CD |
|  |  |  | People 15 years and over who worked in different census subdivision |  |
|  |  |  | Unemployment rate of population 15 years and over with children at home |  |
|  |  |  | People 15 years and over self-employed (unincorporated) without paid help |  |
|  |  |  | People 15 years and over who worked part year or part time |  |
|  | Geographical transitions | Migrants and speaking foreign languages | Movers 1 year ago |  |
|  |  |  | Movers 5 years ago |  |
|  |  |  | Neither English nor French as first official language spoken |  |
|  |  |  | Non-official language spoken in single responses |  |
|  |  |  | English and non-official language in multiple responses |  |
|  |  |  | French and non-official language in multiple responses |  |

**Table 1** (*Continued*)

| **LHT Concept** | **Usual measure in LHT** | **Factor loaded in 1st iteration** | **Variables** | **Final Model** |
| --- | --- | --- | --- | --- |
| - | - | Indigenous | Total aboriginal ancestry population | CD |
|  |  | Visible minority | Total visible minority population | DA |
|  |  |  | Non-official language spoken in single responses | DA |
| - | - | Young children | Age group 0 – 4 years of age | CD; DA |
|  |  |  | Age group 5 – 9 years of age | DA |
| Reproduction | Age of menarche, number of partners, number of children, and interbirth interval | Frequent reproduction | Average size of families | CD; DA |
|  |  |  | Average number of children in families with children | CD; DA |
|  |  |  | Families with 4 persons | CD; DA |
|  |  |  | Families with 5 or more persons | CD; DA |
|  |  | Single parenting | Average family size of one-parent families | CD |
|  |  | Big families | Private households with 4 persons |  |
|  |  |  | Private households with 5 or more persons |  |
|  |  | Recent reproduction | Age group 0 – 4 years of age | CD |

*Note*. Harshness and Unpredictability variables collected from census 2006 and Reproduction variables collected from 2021. Final model indicates whether the variable was a relevant and significant in the models using census division (CD) or dissemination area (DA) sample.

## Table 2

*Formative latent variables assessment of Dissemination Areas model in Study 1.*

| **Latent variables** | | **VIF** | **Weights** | **Loadings** |
| --- | --- | --- | --- | --- |
| Lack of resources | |  |  |  |
|  | Dwellings needing minor repairs | 1.14 | 0.30 | 0.58 |
|  | Dwellings needing major repairs | 1.19 | 0.29 | 0.62 |
|  | Households spending 30%+ of income on rent | 1.17 | 0.72 | 0.90 |
| Divorced or Widowed | |  |  |  |
|  | Divorced | 1.08 | 0.68 | 0.84 |
|  | Widowed | 1.08 | 0.57 | 0.75 |
| Visible minority | |  |  |  |
|  | Visible minority | 2.68 | 0.64 | 0.97 |
|  | Mother tongue is non official languages | 2.68 | 0.42 | 0.92 |
| Young children | |  |  |  |
|  | Children aged 0-4 years | 1.27 | 0.31 | 0.69 |
|  | Children aged 5-9 years | 1.27 | 0.82 | 0.96 |

*Note*. VIF: collinearity assessment. Bootstrapped weights and loadings were all significant (p. < .01) and confidence intervals did not cross zero. Income variable was a single-item variable; therefore, no assessment was made.

## Table 3

*Structural model assessment in Study 1.*

| Dissemination areas model | | | |
| --- | --- | --- | --- |
| **Predictors** | **Frequent reproduction** | | |
|  | VIF | Paths | f² |
| Income | 1.68 | 0.16 | 0.04 |
| Lack of resources | 1.87 | - 0.18 | 0.03 |
| Divorced or Widowed | 1.91 | - 0.17 | 0.03 |
| Visible minority | 1.07 | 0.17 | 0.06 |
| Young children | 1.27 | 0.39 | 0.21 |
| Adj. R² | .49 | | |
| Census divisions model | | | |
| **Predictors** | **Frequent reproduction** | | |
| Unemployed | 1.24 | - 0.38 | 0.62 |
| Young children | 1.24 | 0.66 | 1.82 |
| Adj. R² | .81 | | |
| **Predictors** | **Single parenting** | | |
| Low-income children | 1.60 | 0.34 | 0.20 |
| High rents | 1.49 | - 0.26 | 0.13 |
| Unemployed | 1.44 | - 0.17 | 0.06 |
| Indigenous | 1.62 | 0.30 | 0.15 |
| Young children | 1.73 | 0.42 | 0.28 |
| Adj. R² | .64 | | |

*Note*. VIF: collinearity assessment. Bootstrapped paths were all significant (p. < .01) and confidence intervals did not cross zero.

## Table 4

*Structural model assessment of the tertiles of Children Aged 0-4 years in Study 3.*

|  | Highest percent of children aged 0-4 years | | | |  | Lowest percent of children aged 0-4 years | | | |
| --- | --- | --- | --- | --- | --- | --- | --- | --- | --- |
| **Predictors** | **Frequent reproduction** | | | |  | **Frequent reproduction** | | | |
|  | VIF |  | Paths | f² |  | VIF |  | Paths | f² |
| Unemployed | 1.00 |  | - 0.51*** | 0.52 |  | 1.03 |  | - 0.45*** | 0.47 |
| Young children | 1.00 |  | 0.48*** | 0.36 |  | 1.03 |  | 0.56*** | 0.75 |
| Adj. R² | .51 | | | |  | .59 | | | |
| **Predictors** | **Single parenting** | | | |  | **Single parenting** | | | |
| Low-income children | 2.06 |  | 0.48*** | 0.28 |  | 1.64 |  | 0.35*** | 0.13 |
| High rents | 1.83 |  | - 0.27** | 0.09 |  | 1.59 |  | - 0.49*** | 0.26 |
| Unemployed | 1.87 |  | - 0.21* | 0.06 |  | 1.23 |  | - 0.39*** | 0.21 |
| Indigenous | 2.99 |  | 0.48*** | 0.19 |  | 1.25 |  | 0.13 | 0.03 |
| Young children | 1.57 |  | 0.13 | 0.03 |  | 1.12 |  | 0.40*** | 0.25 |
| Adj. R² | .58 | | | |  | .39 | | | |

*Note*. VIF: collinearity assessment; Bootstrapped paths reported. *: *p ≤ .05, **: p ≤ .01, ***: p ≤ .001*.

## Table 5

*Structural model assessment of the tertiles of Children Aged 5-9 years in Study 3.*

|  | Highest percent of children aged 5-9 years | | | |  | Lowest percent of children aged 5-9 years | | | |
| --- | --- | --- | --- | --- | --- | --- | --- | --- | --- |
| **Predictors** | **Frequent reproduction** | | | |  | **Frequent reproduction** | | | |
|  | VIF |  | Paths | f² |  | VIF |  | Paths | f² |
| Unemployed | 1.03 |  | - 0.54*** | 0.68 |  | 1.07 |  | - 0.37*** | 0.38 |
| Young children | 1.03 |  | 0.46*** | 0.41 |  | 1.07 |  | 0.64*** | 1.12 |
| Adj. R² | .59 | | | |  | .65 | | | |
| **Predictors** | **Single parenting** | | | |  | **Single parenting** | | | |
| Low-income children | 1.62 |  | 0.50*** | 0.37 |  | 1.72 |  | 0.35** | 0.11 |
| High rents | 1.47 |  | - 0.20* | 0.06 |  | 1.68 |  | - 0.47*** | 0.22 |
| Unemployed | 1.57 |  | - 0.25* | 0.10 |  | 1.23 |  | - 0.37*** | 0.19 |
| Indigenous | 1.99 |  | 0.47*** | 0.27 |  | 1.15 |  | 0.12 | 0.02 |
| Young children | 1.21 |  | 0.18* | 0.07 |  | 1.16 |  | 0.48*** | 0.33 |
| Adj. R² | .56 | | | |  | .37 | | | |

*Note*. VIF: collinearity assessment; Bootstrapped paths reported. *: *p ≤ .05, **: p ≤ .01, ***: p ≤ .001*.

## Table 6

*Structural model assessment of the tertiles of Visible Minorities in Study 4.*

|  | Highest percent of Visible minorities | | | |  | Lowest percent of Visible minorities | | | |
| --- | --- | --- | --- | --- | --- | --- | --- | --- | --- |
| **Predictors** | **Frequent reproduction** | | | |  | **Frequent reproduction** | | | |
|  | VIF |  | Paths | f² |  | VIF |  | Paths | f² |
| Unemployed | 1.08 |  | - 0.41*** | 0.69 |  | 1.2 |  | - 0.42*** | 0.93 |
| Young children | 1.08 |  | 0.69*** | 2.07 |  | 1.2 |  | 0.66*** | 2.27 |
| Adj. R² | .79 | | | |  | .84 | | | |
| **Predictors** | **Single parenting** | | | |  | **Single parenting** | | | |
| Low-income children | 1.87 |  | 0.42*** | 0.25 |  | 1.41 |  | 0.31*** | 0.23 |
| High rents | 1.98 |  | - 0.35*** | 0.16 |  | 1.24 |  | - 0.11 | 0.03 |
| Unemployed | 1.70 |  | - 0.18* | 0.05 |  | 1.44 |  | - 0.13 | 0.03 |
| Indigenous | 1.79 |  | 0.20* | 0.06 |  | 1.97 |  | 0.22** | 0.07 |
| Young children | 1.57 |  | 0.60*** | 0.63 |  | 1.97 |  | 0.52*** | 0.45 |
| Adj. R² | .62 | | | |  | .66 | | | |

*Note.* VIF: collinearity assessment; Bootstrapped paths reported. *: *p ≤ .05, **: p ≤ .01, ***: p ≤ .001.*

## Figure 1

*Decision-making process in the PLS-SEM analysis*


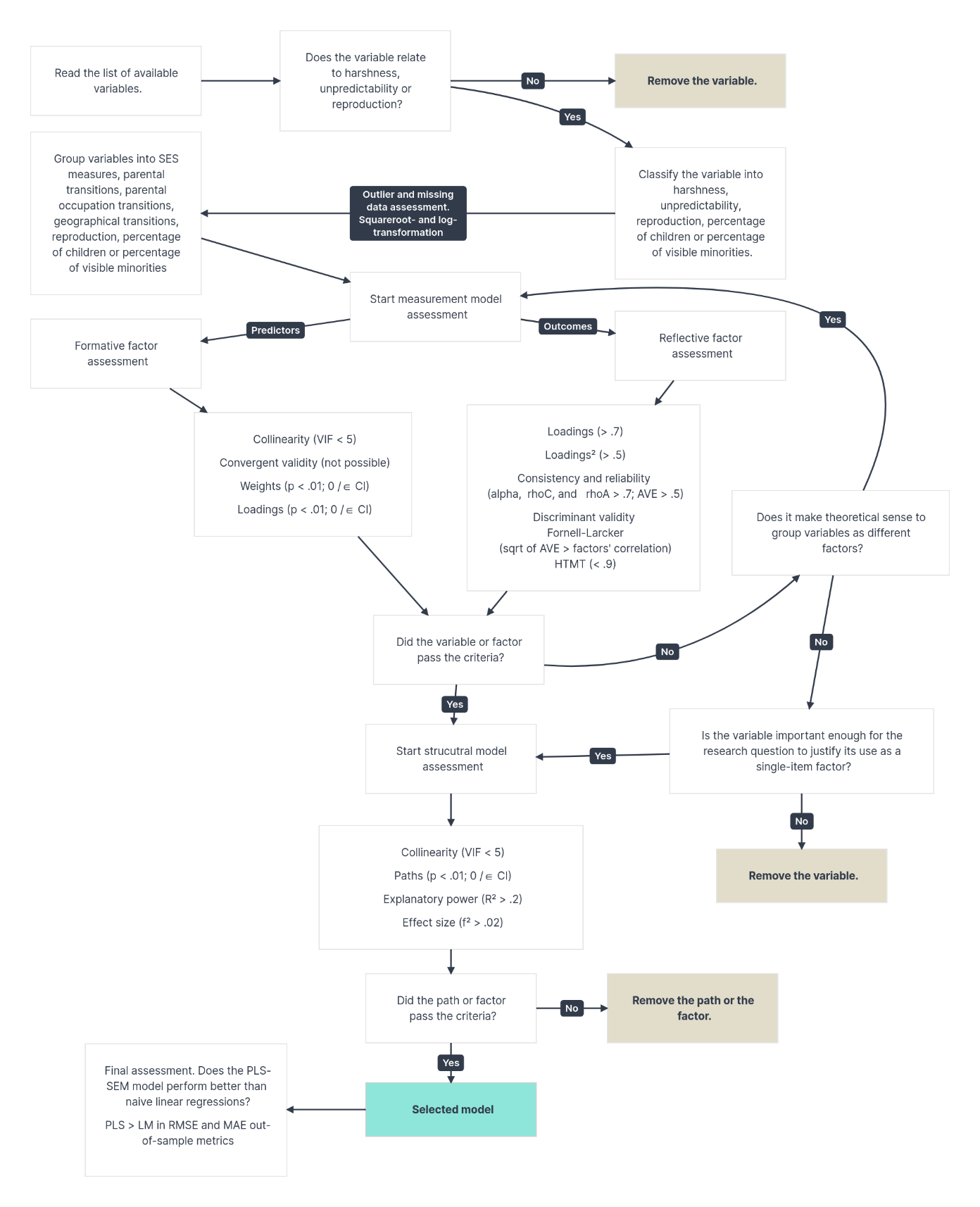


*Note.* AVE : Average variance extracted; HTMT: Heterotrait-Monotrait Ratio.

## Figure 2

*Proportion of young children, rates of indigeneity in the population, and socioeconomic factors predict* *early reproduction in Dissemination Areas and Census Divisions.*

| DA Model |  | CD model |
| --- | --- | --- |
| 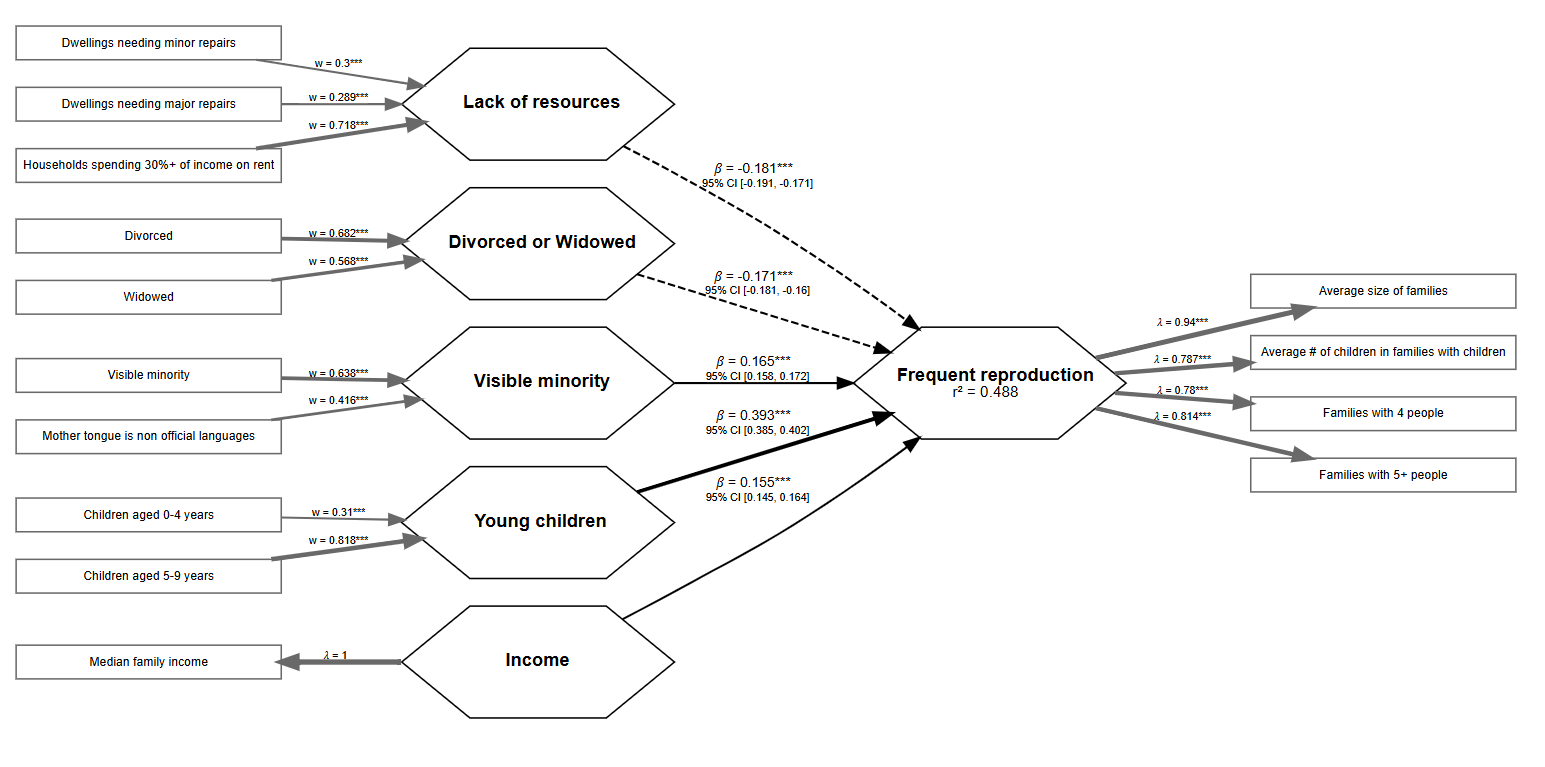 |  | 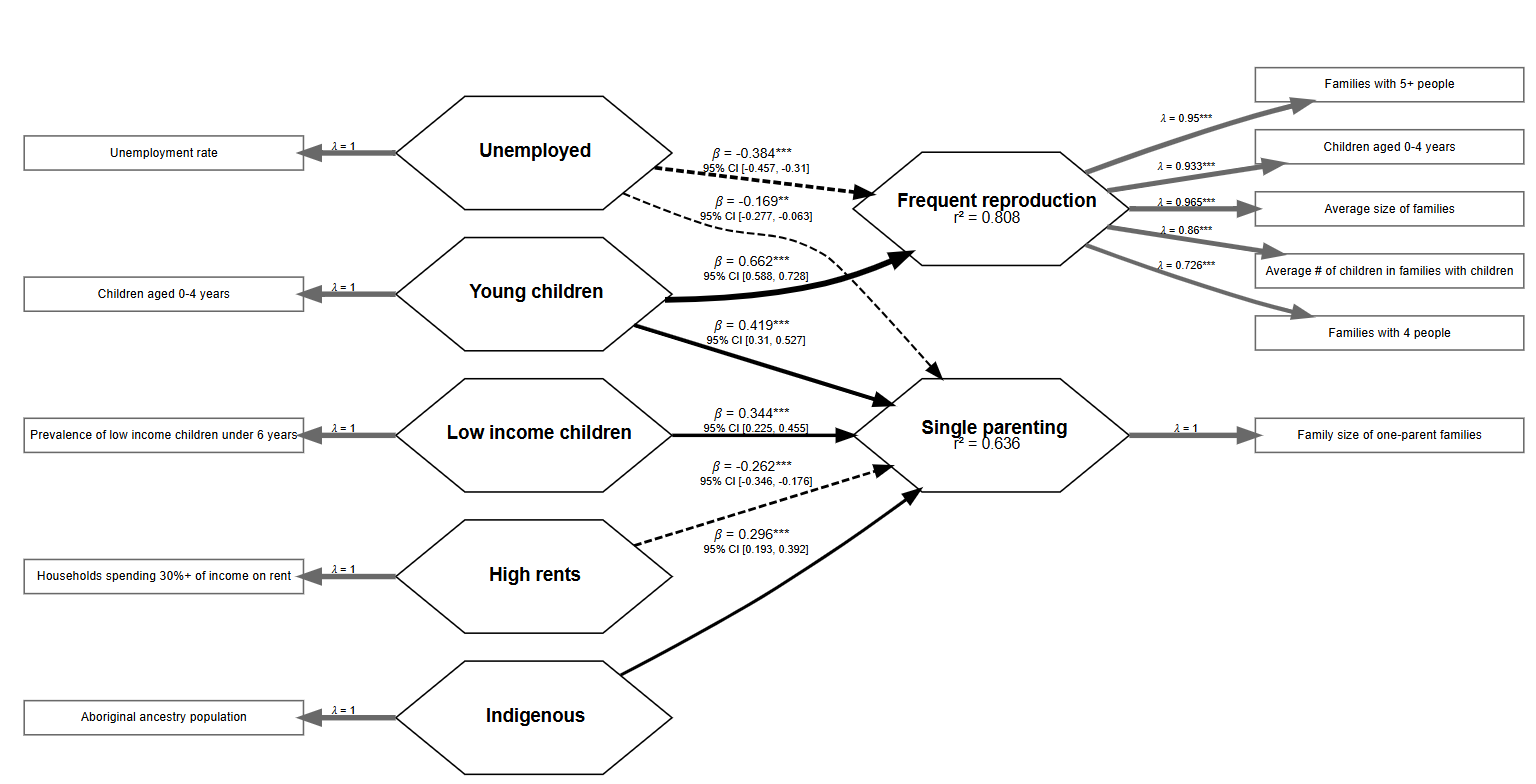 |

Note. Figure created in R using seminr package. Hexagons represent latent variables and rectangles represent items from census. Predictors are formative latent variables using census data in 2006 and outcomes are reflective latent variables using census data in 2021. DA = Dissemination Areas; CD = Census Divisions; W: variable’s weights, λ: variable’s loadings, β; path’s beta coefficients; **: *p* ≤ .01, ***: *p* ≤ .001.

## Figure 3

*Later harshness and unpredictability are poor predictors of previous measures of early reproduction.*


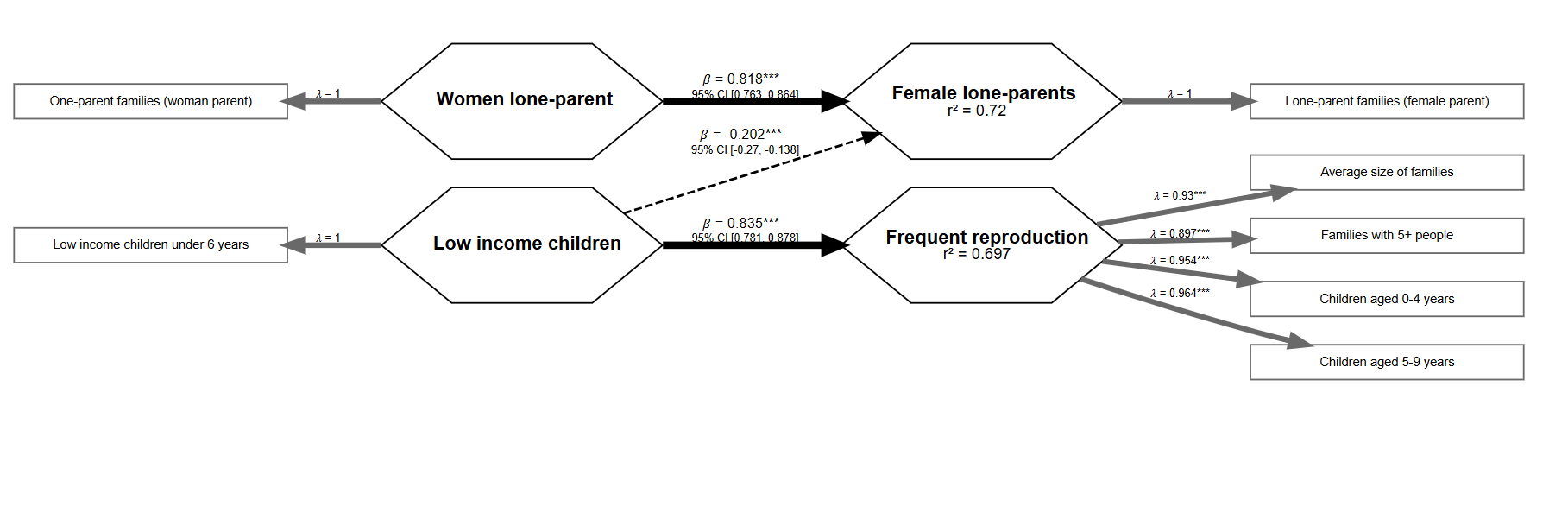


*Note*. Figure created in R using seminr package. Hexagons represent latent variables and rectangles represent items from census. Predictors are formative latent variables using census data in 2006 and outcomes are reflective latent variables using census data in 2021. DA = Dissemination Areas; CD = Census Divisions; W: variable’s weights, λ: variable’s loadings, β; path’s beta coefficients; **: *p* ≤ .01, ***: *p* ≤ .001.
